# T Cell Repertoire Diversity Is Decreased in Type 1 Diabetes Patients

**DOI:** 10.1016/j.gpb.2016.10.003

**Published:** 2016-12-24

**Authors:** Yin Tong, Zhoufang Li, Hua Zhang, Ligang Xia, Meng Zhang, Ying Xu, Zhanhui Wang, Michael W. Deem, Xiaojuan Sun, Jiankui He

**Affiliations:** 1 Department of Biology, South University of Science and Technology of China, Shenzhen 518055, China; 2 Department of Endocrinology, Zhujiang Hospital, Southern Medical University, Guangzhou 510280, China; 3 Department of Gastrointestinal Surgery, Shenzhen People's Hospital, the Second Clinical Medical College of Jinan University, Shenzhen 518020, China; 4 Department of Infectious Diseases and Hepatology Unit, Nanfang Hospital, Southern Medical University, Guangzhou 510515, China; 5 Departments of Bioengineering and Physics & Astronomy, Rice University, Houston, TX 77005, USA; 6 Shenzhen Tumor Immune-Gene Therapy Clinical Application Engineering Lab, Biobank of the Second People's Hospital, the First Affiliated Hospital of Shenzhen University, Shenzhen 518035, China

**Keywords:** Diversity, High-throughput sequencing, Immune repertoire, T cell receptor, Type 1 diabetes

## Abstract

**Type 1 diabetes** mellitus (T1D) is an immune-mediated disease. The autoreactive T cells in T1D patients attack and destroy their own pancreatic cells. In order to systematically investigate the potential autoreactive **T cell receptors** (TCRs), we used a high-throughput **immune repertoire** sequencing technique to profile the spectrum of TCRs in individual T1D patients and controls. We sequenced the T cell repertoire of nine T1D patients, four type 2 diabetes (T2D) patients, and six nondiabetic controls. The **diversity** of the T cell repertoire in T1D patients was significantly decreased in comparison with T2D patients (*P* = 7.0E−08 for CD4^+^ T cells, *P* = 1.4E−04 for CD8^+^ T cells) and nondiabetic controls (*P* = 2.7E−09 for CD4^+^ T cells, *P* = 7.6E−06 for CD8^+^ T cells). Moreover, T1D patients had significantly more highly-expanded T cell clones than T2D patients (*P* = 5.2E−06 for CD4^+^ T cells, *P* = 1.9E−07 for CD8^+^ T cells) and nondiabetic controls (*P* = 1.7E−07 for CD4^+^ T cells, *P* = 3.3E−03 for CD8^+^ T cells). Furthermore, we identified a group of highly-expanded T cell receptor clones that are shared by more than two T1D patients. Although further validation in larger cohorts is needed, our data suggest that T cell receptor diversity measurements may become a valuable tool in investigating diabetes, such as using the diversity as an index to distinguish different types of diabetes.

## Introduction

Type 1 diabetes mellitus (T1D) is an autoimmune disease characterized by infiltration of leukocytes into the islets of the pancreas, resulting in progressive pancreatic β-cell destruction and loss of insulin production [1], [2], [3]. The infiltrating cells are a heterogeneous population, composed mainly of CD4^+^ and CD8^+^ T lymphocytes, as well as some B lymphocytes, macrophages, and dendritic cells [4], [5], [6], [7]. The most direct evidence of the pathogenic role of T cells in T1D is from the biobreeding rat and the nonobese diabetic (NOD) mouse models [8], [9]. Extensive studies in mouse models have demonstrated that T cells play crucial roles in the pathogenesis of T1D, as the disease can be transferred by T cell clones or a heterogeneous T cell population [10], [11], [12]. For example, Wicker et al. transferred splenocytes of NOD mice to young mice. Consequently these recipient mice develop diabetic at a higher frequency and at a younger age than their controls [10]. Notably, CD4^+^ and CD8^+^ T cell subpopulations play different roles in the process of T1D initiation. CD4^+^ T cells mostly recognize insulin and are the main cellular effectors, whereas CD8^+^ cytotoxic T cells recognize peptide epitopes presented on the β cell surface and directly contribute to β cell death [13], [14].

The diversity of the T cell immune repertoire is critical in maintaining an effective immune response, and decreased diversity of the T cell immune repertoire has been linked to several autoimmune diseases, such as rheumatoid arthritis [15] and multiple sclerosis [16], [17] and aging [18].

Over the past two decades, researchers have shown that restricted T cell expansion and reduced T cell diversity in pancreatic islets is a common phenomenon in T1D. In early work, the biased usage of some T cell receptor (TCR) gene segments was found in islet-infiltrated T cells [19]. In 2009, Li et al. [19] performed single-cell PCR to analyze the TCR sequences of 218 T cells in NOD mice and discovered a restricted repertoire dominated by one or two clones, suggesting the monoclonal expansion of T cells in pancreatic islets of NOD mice [20]. In 2011, another group cloned 139 different TCR complementarity-determining region 3 (CDR3) sequences and revealed the monoclonal expansion of T cells in human pancreatic islets [21]. These studies mainly used traditional cloning and sequencing methods to identify TCRs when examining the T cell composition in the islet infiltrate in T1D. However, this traditional approach has some limitations in studying TCR restriction. For example, the size of the TCR repertoire in a human being is estimated to be as many as 10^7^ clones, whereas current cloning and sequencing can only identify a few hundreds of sequences [22], which only account for a tiny fraction of the total repertoire. Therefore, the overall diversity of the TCR repertoire in T1D patients has not been studied yet, due to the technical limitations.

Here, we applied a recently-developed high-throughput immune repertoire sequencing technique to investigate the T cell immune repertoire diversity in T1D patients. Immune repertoire sequencing is a powerful technique which is able to sequence millions of TCR or B cell receptor sequences in parallel in a single sample [23], [24], [25], [26]. We sequenced an average of 10^5^ TCR sequences per sample, which covers all the dominant TCR clones in the sample. By analyzing a large number of TCR sequences, we characterized the overall diversity of the immune repertoire, V gene usage bias, VDJ recombination pattern, and CDR3 length distribution in both CD4^+^ and CD8^+^ T cell subtypes. We also identified common T cell clones that are shared by multiple T1D patients. Considering TCRs and human leukocyte antigen (HLA) are closely related, we also investigated HLA genotyping in some T1D patients.

## Results

Nine T1D patients, four T2D patients, and six nondiabetic controls were recruited for this study. Peripheral blood mononuclear cells (PBMCs) were isolated from blood samples to sort CD4^+^ and CD8^+^ T cells. Multiplex PCR was performed to amplify the CDR3 regions for construction of libraries, which were sequenced on the Illumina HiSeq 2000/Miseq platform. Sequencing reads were analyzed using our in-house bioinformatics pipeline and the online ImMunoGeneTics (IMGT)/HighV-QUSET tool [27], [28].

A total of 16,376,727 merged sequencing reads were obtained from raw sequencing data. Sequencing reads were aligned against the reference sequences of genes encoding human T cell receptor beta variable (TRBV), diversity (TRBD), and joining (TRBJ) [29]. Reads with a high identity score (>70%) were selected and identified as TCRβ chain sequences. As a result, we identified 4,875,520 *TCR*β sequencing reads from control samples, 4,738,895 reads from T2D samples, and 1,500,011 reads from T1D samples, respectively.

### Relatively more highly-expanded T cell clones found in T1D

**Figure 1:**
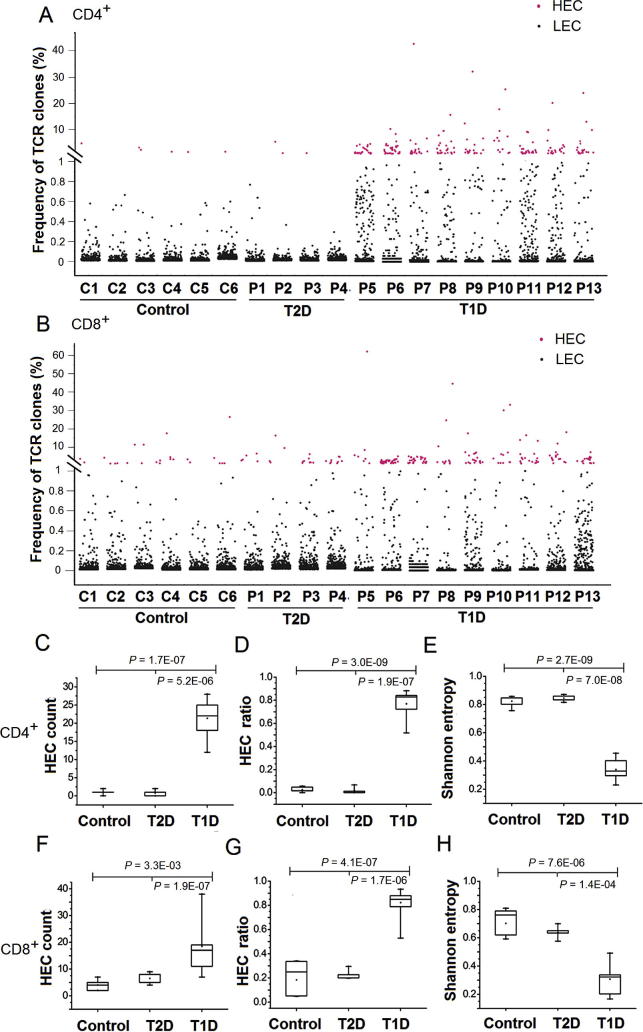
**HECs and diversity in T1D** Scatterplot showing distribution of all TCR clone sequences from CD4^+^ (**A**) and CD8^+^ T cells (**B**) of T1D, T2D, and control samples. Each data point represents a CDR3 clone. Y axis shows the frequency of each clone observed in different samples, expressed as the number of a specific TCR clone against the total number of CDR3 sequences in respective samples (%). HECs (frequency ⩾1%) are shown in red and LECs (frequency <1%) are shown in black. **C.**–**H.** Statistical analysis of TCR clones of CD4^+^ and CD8^+^ T cells using Shannon entropy, HEC number, and HEC ratio in T1D, T2D, and nondiabetic control samples. HEC counts indicates the sum of HECs in a sample, and HEC ratio indicates the ratio of total sequence counts of HECs relative to the total sequence counts of the sample. All statistical analyses were performed with unpaired two-tailed *t*-test using R software package. CDR, complementarity-determining region 3; HEC, highly-expanded clone; LEC, lowly-expanded clone; T1D, type 1 diabetes mellitus; T2D, type 2 diabetes mellitus; TCR, T cell receptor.

TCR clones with frequency ⩾1% of total reads in a sample were defined as highly-expanded clones (HECs). As shown in Figure 1 and Figure S1, T1D patients have more HECs compared to T2D and control samples for both CD4^+^ and CD8^+^ T cells (Figure1A and B). In the CD4^+^ T cell population, T1D patients have 22 HECs (median, range of 12–28), which is much higher than those in T2D (median of 1 HEC, range 0–2) and control samples (Figure1C). As shown in Figure1D, HECs accounted for 77% of total sequencing reads in the T1D patients (median, range 52%−88%), which is much higher than those in T2D patients (median of 3.9%, range 0–6.8%) and controls (median of 1.7%, range 0–5.7%). Similar trend was also observed in the CD8^+^ T cell population (Figure1F and G).

We applied a normalized Shannon entropy index to quantitatively measure the diversity of the entire TCR repertoire in different groups [30]. The normalized Shannon entropy index ranges from 0 to 1, in which “1” indicates the most diversity and “0” indicates no diversity at all. The normalized Shannon entropy of T1D samples was significantly lower than that of T2D and control samples for both CD4^+^ (Figure1E) and CD8^+^ T cells (Figure1H). These data indicate that the overall diversity of the entire TCR repertoire of T1D patients is significantly decreased compared with T2D and nondiabetic controls.

Collectively, these findings show that although thousands of TCR clones were observed in T1D, the TCR repertoire of T1D is dominated by a few HECs. Our results are consistent with previous studies using T1D mouse models, in which HECs are frequently observed in islet-infiltrating T cells [31], [32], [33]. The significant difference in the number and percentage of HECs between T1D and T2D samples indicates that the quantification of HECs and diversity could be a potential indicator of T1D.

### Shared TCR clones among T1D samples

**Figure 2:**
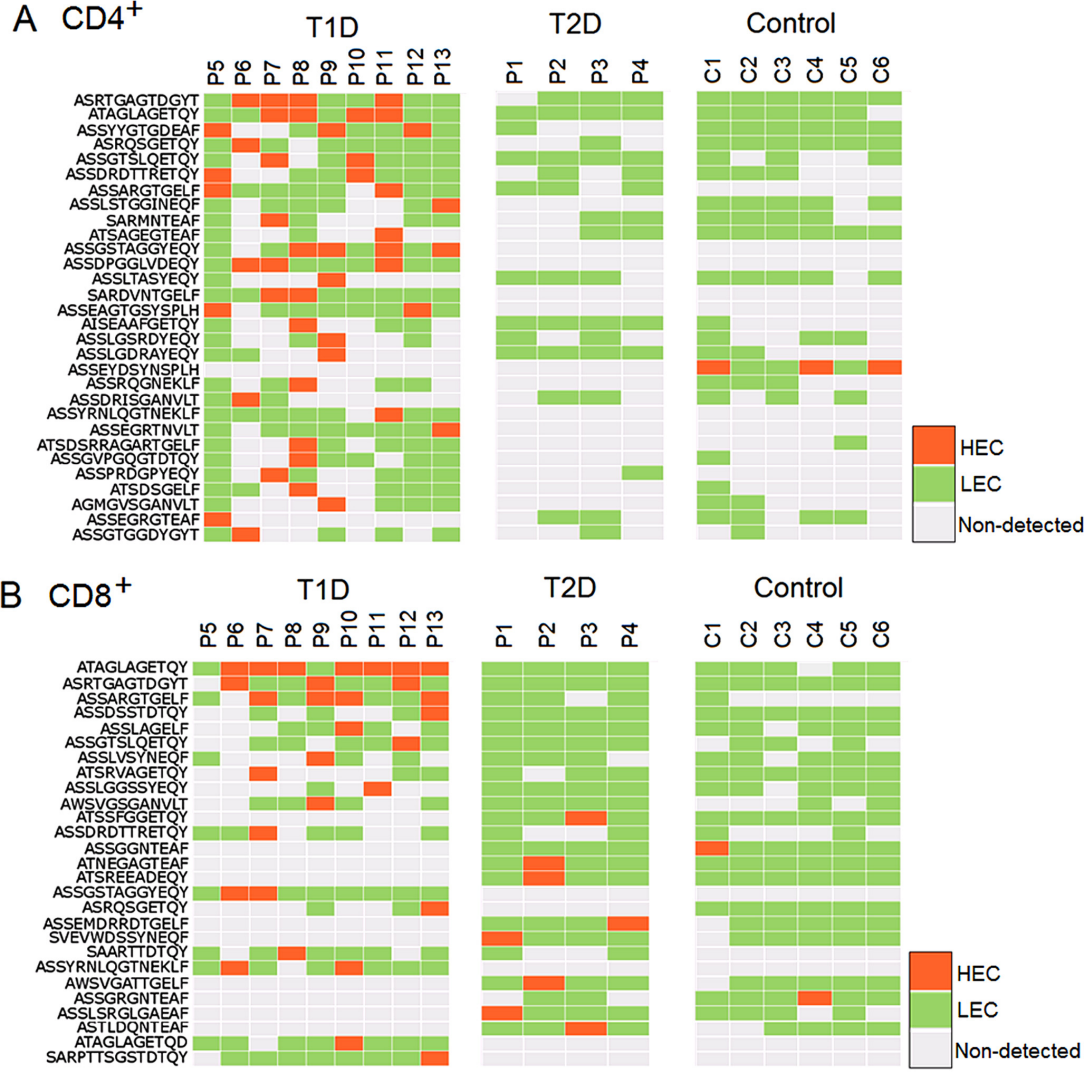
**Shared HECs are detected in different T1D patients** HECs that are observed in multiple T1D patients are shown for CD4^+^ (**A**) and CD8^+^ (**B**) T cells. Only HECs significantly shared by multiple patients are shown, the complete list of shared HECs can be found in Figure S5. Orange and green represent HECs (⩾1% of total reads) and LECs (<1% of total reads), respectively, while gray represents sequences not found in the sample. HEC, highly-expanded clone; LEC, lowly-expanded clone; T1D, type 1 diabetes mellitus; T2D, type 2 diabetes mellitus.

The second finding in our study is that several HECs observed in one T1D patient were also observed in other T1D patients. In CD4^+^ T cells, 4 HECs are expressed in all T1D samples tested and 52 HECs are detected in over half of the samples. In CD8^+^ T cells, 2 HECs are expressed in all T1D samples tested and 36 HECs are detected in over half of the samples. These data suggest that T1D patients share some common HECs. The shared or common T cells are of long-term interest both in health and disease [34]. To investigate the possible common TCRs that are shared in T1D pathogenesis, we analyzed the CDR3 amino acid sequence of all HECs in all the samples tested. The HECs were then ranked according to the number of patients sharing these HECs (Figure 2). As a result, we observed two types of HECs. The first type of HECs were HECs shared in T1D samples, which are present but not identified as HECs in T2D or control samples. For example, the TCR CDR3 sequence ASRTGAGTDGYT was observed as a HEC in CD4^+^ T cells of four T1D patients (first row, shown in orange in the left panel, Figure2A). Although this sequence is also present in T2D and control samples, it is not classified as a HEC (shown in gray or green) in any T2D (middle panel) or control samples (right panel, Figure2A). The second type of HEC is unique to T1D samples, which are not observed in any T2D or control sample. For example, the TCR clone CDR3 sequence ASSEAGTGSYSPLH is classified as a HEC in two T1D patients (15th row, shown in orange in the left panel), but it is not present in any of the T2D or control samples (shown in gray in the middle and right panels, Figure2A). Among the total 185 observed CDR3 HECs (by amino acid sequences) in CD4^+^ T cells, the first type accounts for 32.4%, whereas the remaining 67.3% falls into the second category, which is only observed in T1D samples.

In CD8^+^ T cells, there are totally 203 observed CDR3 amino acid HECs, including 43.8% for the first type and 56.2% for the second type (Figure2B). It should be noted that we only have a limited number of control and T2D samples included in this study. Some of the second type HECs may turn out to be the first type HECs, if more T2D and control samples could be sequenced in future.

### V and J gene usage in T1D samples

**Figure 3:**
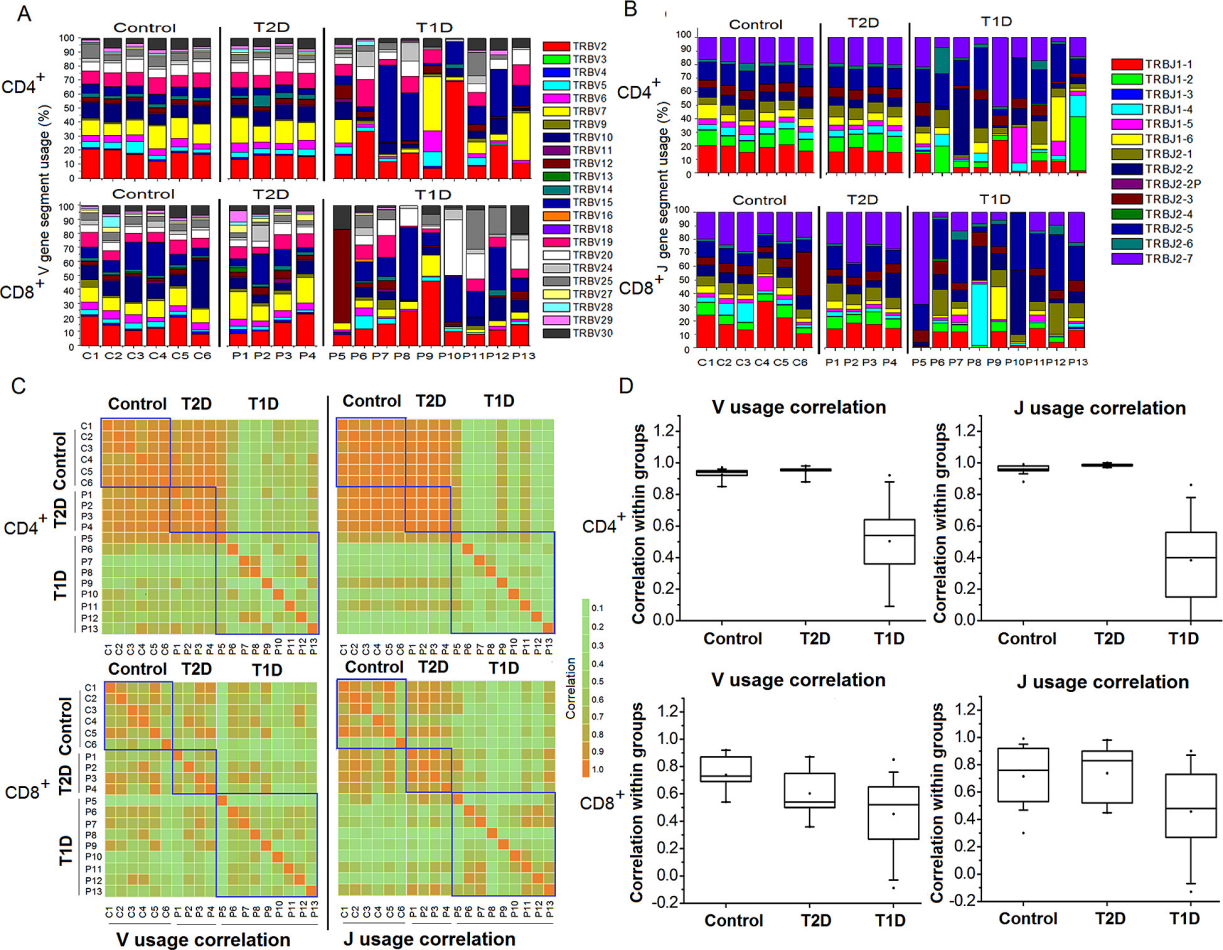
**V and J gene usage analysis in T1D, T2D, and control samplesA.** Stacked bar chart for the frequency of 23 V gene family usages in T1D, T2D, and control samples expressed as percentages. The V gene families are color-coded as shown in the figure. **B.** Stacked bar chart for the frequency of 14 J gene family usages in T1D, T2D, and control samples expressed as percentages. The J gene families are color-coded as shown in the figure. **C.** Pairwise correlation of V/J gene family usage between different samples. Pearson’s correlations were calculated for the frequency of 23 V gene families (left panel) and 14 J gene families (right panel). The intensity of correlation was color-coded as shown in the figure. **D.** Box plot of pairwise correlation of samples in the same group. T1D, type 1 diabetes mellitus; T2D, type 2 diabetes mellitus. Individual points in the box mean the outliers.

To identify potential V or J gene usage bias in T1D samples, we then investigated the germline gene usage in T1D and T2D patient as well as nondiabetic control samples (Figure 3). Our data showed that the V gene family usage pattern was very similar between the control and T2D patient samples. However, the V gene family usage patterns of T1D patients were highly heterogeneous in CD4^+^ T cells (Figure3A). This heterogeneity may be explained by the observation that different T1D patients have different HECs (Figure 2). For the CD8^+^ T cells, T1D, T2D, and control samples all display a heterogeneous pattern (Figure3A). Similar phenomenon is also observed in J gene usage for both CD4^+^ and CD8^+^ T cells (Figure3B). We then performed statistical analysis to identify any V gene families that were significantly overused in T1D. Consequently, we observed V gene usage biases in different samples. The V and J gene usage pattern of control and T2D samples were highly correlated, particularly in CD4^+^ T cells (Figure3C). The average correlation between any 2 samples in control and T2D groups was 0.97 (Figure3D). Conversely, the T1D samples displayed less correlation in V and J gene usage pattern, probably due to patient-specific clonal expansion.

**Figure 4:**
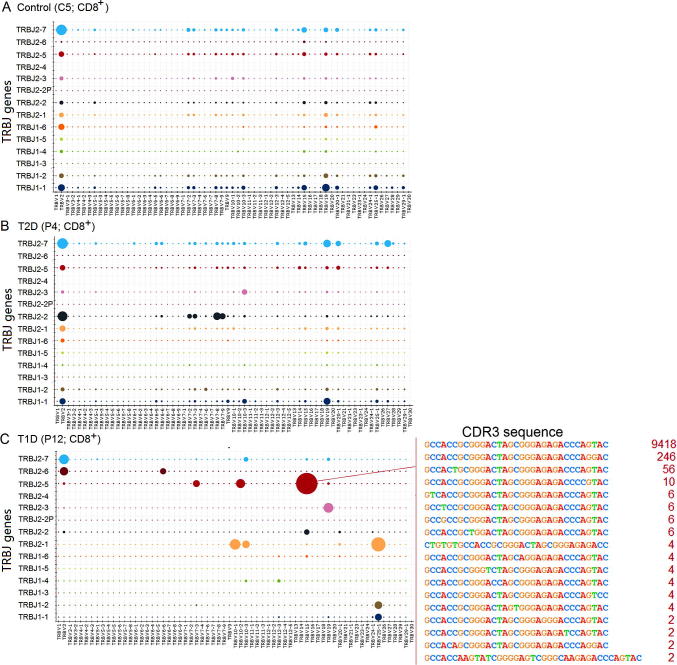
**Entire VJ repertoire in CD8^+^ cells for representative samples C5, P4, and P12** Entire VJ repertoire in CD8^+^ cells for samples C5 (**A**), P4 (**B**), and P12 (**C**) was plotted, which represent nondiabetic control, T2D and T1D patients, respectively. X and Y axes list all possible V gene and J usages, respectively, while each point in the 2-dimensional space represents a unique VJ combination. The size of the sphere at each point corresponds to the number of reads matching that particular VJ combination. CDR3 sequences and read number of dominant clones (TRBV15–TRBJ2-5) were also show in panel C. Other samples are listed in Figure S2. T1D, type 1 diabetes mellitus; T2D, type 2 diabetes mellitus.

The skewed clonotype composition in T1D samples is also observed in the global VJ combination. The global VJ combination usage of three representative samples is shown in Figure 4. CD8^+^ T cells in the control (C5) and T2D (P4) samples display high diversity of immune repertoire that is represented by a broad usage of VJ combinations (Figure4A and B). On the other hand, the T1D sample (P12) has a dominant VJ combination (TRBV15 and TRBJ2-5), which accounts for 27.5% of total reads. The HEC sequence which corresponds to protein sequence ATAGLAGETQY is present in this dominant VJ combination, indicating a strong clonal expansion (Figure4C). The global VJ combination usage of all samples is shown in Figure S2. The comprehensive analysis of V gene usage and VJ combinations in T1D samples is shown in Figures S3 and S4, respectively.

### Skewed CDR3 length distribution in T1D

**Figure 5:**
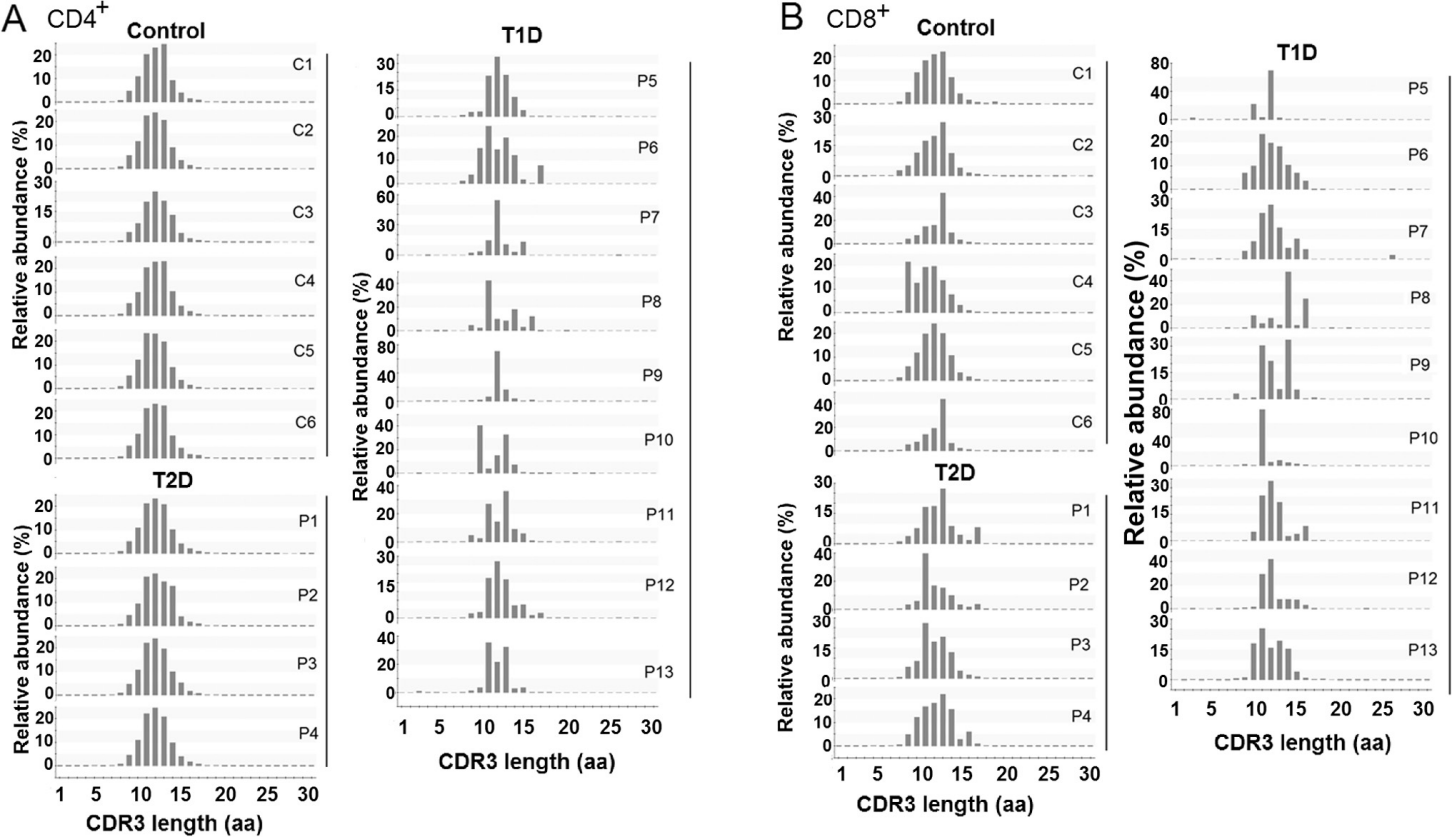
**CDR3 amino acid length distribution** Histogram of CDR3 length distribution in CD4^+^ and CD8^+^ T cells from control, T2D, and T1D samples was calculated from the sequencing reads and shown in **A** and **B**, respectively.

CDR3 length influences the structure of TCRs, in which one amino acid differences can lead to conformational remodeling of the receptor [22]. Hence, we perform the statistical analysis of the length distribution of CDR3 here. The CDR3β length distribution of T1D displays a distorted pattern in both CD4^+^ and CD8^+^ T cells (Figure 5). The T2D and control samples have a Gaussian distribution of CDR3β length. However, distorted distribution of CDR3β length is observed in the T1D samples. This provides further evidence that the immune repertoires of T1D patients are skewed, probably owing to patient-specific clonal expansion.

### PCR amplification bias validation

**Figure 6:**
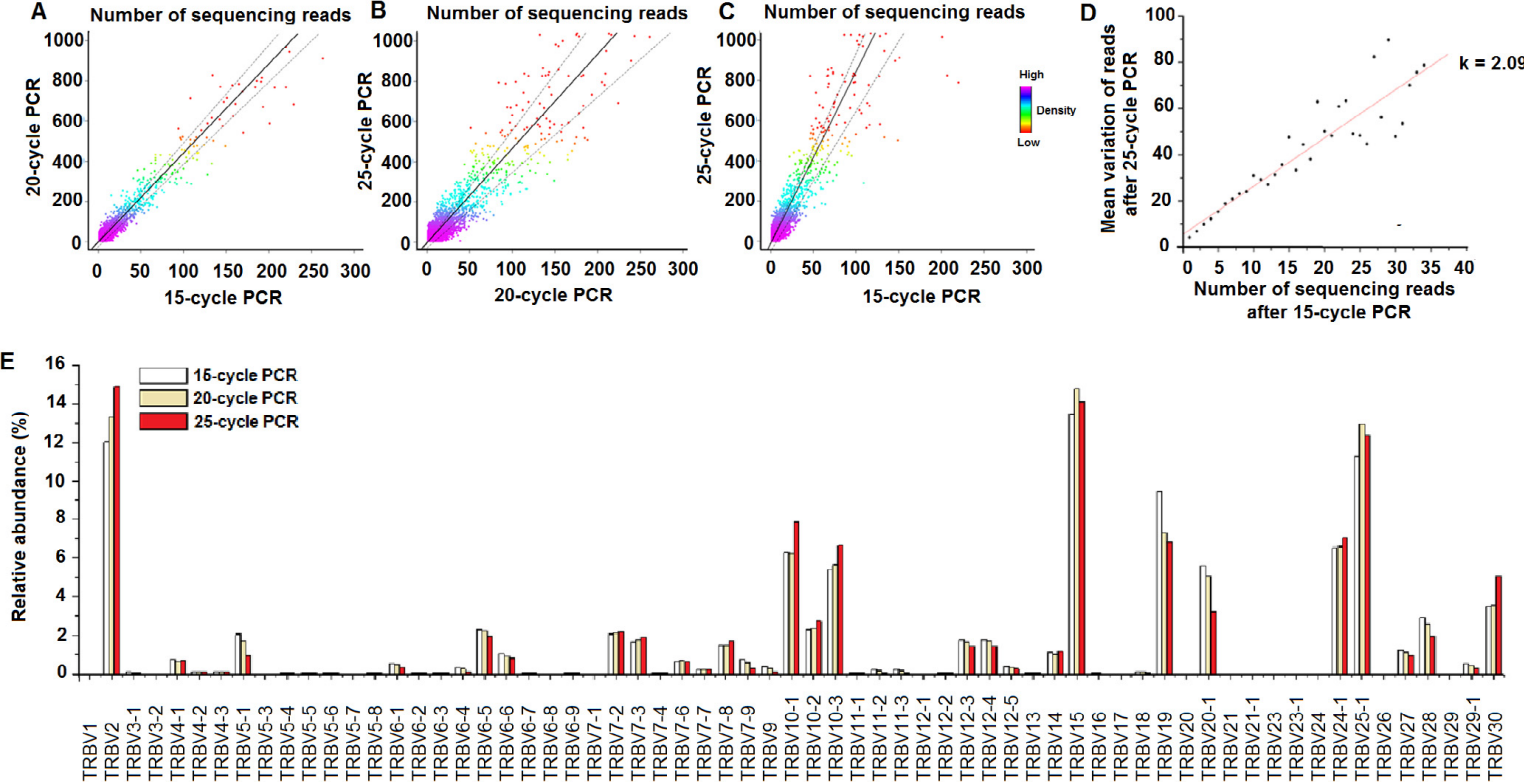
**PCR bias assessmentA.**–**C.** Assessment of PCR bias. Each point represents a single unique CDR3 sequence, which is plotted according to the number of reads observed in the 15-cycle, 20-cycle, and 25-cycle PCR amplifications. Density of sequences for each dot in the plot is color coded, with red the lowest density and purple the highest. Solid line represents a linear regression of the data, and the dotted lines represent the standard deviations. **D.** The mean variation between the number of reads after 15-cycle PCR and 25-cycle PCR is plotted in this figure. Data points indicate the mean variation of read number in 25-cycle PCR is represent, and solid line represents a linear regression of the data. The CDR3 sequences observed after 15-cycle PCR with the same frequencies are observed with different frequencies after 25-cycle PCR. The mean variation in the number of observations represents the PCR bias, *i.e.*, the same number of sequences can be amplified to more or less extent. **E.** The frequency of each *TRBV* gene segments after 15, 20, and 25 cycles of PCR amplification.

As the full repertoire of TCR is amplified by multiple PCR primers, differences in amplification efficiency can affect the real amount of individual TCRs. So first of all, we need to validate the primers amplification level to make sure the designed primer set have similar amplification efficacy. We validated the *TCR*β primers of their amplification efficiency using a method developed by Robins et al. [35] with detailed procedure described previously [22]. We amplified the same DNA extracted from T cells of one individual by using the same primer set with 15, 20 and 25 PCR cycles respectively, and compared the number of reads for the same TCR clones (Figure 6). We observed a linear correlation between the numbers of reads obtained (106,999 reads for the 15-cycle amplification and 703,443 reads for the 25-cycle amplification; Figure6A–C). For sequences observed with a given read number in the 15-cycle amplification, the variance of read number at 25-cycle amplification could be due to the PCR bias. As shown in Figure6D, the regression coefficient *k* = 2.09 represents the bias value after 10 PCR cycles from 15-cycle amplification to 25-cycle amplification. Therefore, a bias of average magnitude 1.07 was introduced in each PCR cycle, eventually resulting in a total accumulated variation about 2.09-fold after 10 more cycles (1.076^10^ = 2.09). However, the abundance of V gene usage of the 20-cycle amplification and that of the 25-cycle amplification are very similar, with 15% deviation for the top 20 V gene families on average. This result indicates that the efficiency of the primers used are very close to each other, and the PCR bias are thus random events (Figure6E).

## Discussion

We present here quantitative measurement of the TCR repertoire of T1D and T2D patients as well as nondiabetic controls. We observed a significant increase in highly-expanded TCR clones and decrease of TCR diversity in T1D patients. The increase in HECs is also observed in other autoimmune diseases, including systemic lupus erythematosus and rheumatoid arthritis [24], indicating that the increase of HECs may be a common phenomenon in autoimmune diseases. Our data suggest that the HECs could be used to distinguish autoimmune T1D from T2D and nondiabetic controls. Nonetheless, it should be noted that the number of patients in the current study is far from enough to distinguish the autoreactive T cells that are related to diabetes. To create the common clone and apply these findings to T1D diagnosis, validation in a larger cohort with more patients is required. Here, our pilot study illustrates the application of the immune repertoire sequencing in screening the candidate common clones.

Interestingly, we observed the sharing of HECs between different T1D patients in this study. Circulation of islet-infiltrating autoreactive T cells responsible for T1D onset in the peripheral blood had also been noticed by several other groups [36], [37], [38]. Although functional assays were not performed to verify the targets of HECs in this study, a few HECs were shared by more than 3 T1D patients, suggesting that they may be derived from the autoreactive TCR immune response to common autoantigens in T1D. The combination of high-throughput screening and functional assay may facilitate the identification of autoreactive T cells. This study will facilitate the understanding of the pathogenesis of auto immune diseases, and help developing potential markers to diagnose the preclinical autoimmune disorders [39]. Once the HEC library in T1D identified in this study is validated by immunophenotyping or other methods, we can use mass spectrometry to identify the associated autoantigens that are not described previously.

The shared HECs between T1D and controls may illustrate the origin of autoreactive TCRs. Two theoretical models could explain the origin of autoreactive TCRs [40], [41]. The first model hypothesizes that the high-affinity T cells to the autoantigens in T1D patients may bypass the deletion process in thymus, migrate to the periphery, and become autoreactive T cells [40]. According to this model, the autoreactive T cells in T1D should only exist in T1D patients and should not be observed in non-T1D patients. The second model proposed that both healthy individuals and T1D patients have autoreactive T cells, whereas only autoreactive T cells in T1D are triggered and react to autoantigen [41]. From our data, we observed the co-existence of two types of HECs. The first type of HECs observed in T1D exists also in controls, whereas the second type of HECs is observed in T1D patients only. Our results may thus suggest the co-existence of both models. There are slight differences in CD4^+^ and CD8^+^ T cells with regard to the HEC numbers, V gene usage pattern, and CDR3 length distribution, which may be associated with the different biological functions of these two types of T cells [42], [43]. However, the immune system is very sensitive to the environment and infection, *e.g.*, by flu in the past 2–3 weeks, which would result in some dominant clones [25]. Although we obtained HECs that are shared in most T1D patients, careful validation need to be done before we can draw the conclusion that these HECs are really T1D-associated.

HLA/peptide complex and TCR binding determines the specificity of immune response [44], [45], [46]. HLA genotyping in 6 T1D patients (Table S1) indicated that alleles such as A*24:02, B*58:01:01, C*03:02, DRB1*03:01, DRB1*09:01:02, DQB1*03:02:01, and DQB1*02:01:01 are frequently expressed in these patients, suggesting that these types of HLAs may play roles in T1D biogenesis.

The immune repertoire sequencing in the T1D samples opens a new vision for investigation of T1D and related immune disorders. However, at present, the work is still on the initial stage. Considering the mismatch in age and gender of subjects between different groups may lead to bias in data interpretation, a larger patient size is needed to achieve a more solid conclusion. With more diabetic samples included in the similar work, as well as the follow-up experimental validation, animal model, and clinical data, the immune repertoire sequencing can provide new diagnostic and therapeutic markers for T1D.

In conclusion, deep sequencing of the CDR3 region of TCR populations using immune-repertoire sequencing can be a powerful tool to access the majority of TCR diversities in peripheral blood of both diabetic patients and controls. The large volume of TCR sequencing data allows us to obtain a snapshot of the entire repertoire. By quantitatively measuring the diversity of the immune repertoire, immune repertoire sequencing maybe helpful to narrow down the potential CDR3 sequences that are related to the autoreactive T cells in T1D.

## Methods

### Ethics

The study was performed according to the principle of declaration of Department of Endocrinology, Zhujiang Hospital of Southern Medical University, China. Study protocol was approved by the medical ethics committee of this university. All participants gave written informed consent.

### Patients

Nine T1D patients, 4 T2D patients, and 6 nondiabetic controls were included in this study. All diabetic patients fulfilled the classification criteria for either T1D or T2D respectively (Table S2) [47]. We acquired 10 ml of peripheral blood from all subjects. The glucose level and C-peptide concentration were calculated according to the methods recommended by the WHO (https://www.staff.ncl.ac.uk/philip.home/w-ho_dmc.htm). To eliminate the potential influence from other autoimmune disease or infection, we only selected the candidate samples from patients without other autoimmune diseases or infections in the past 4 weeks.

### HLA genotyping

Five major HLA types, namely, HLA-A, HLA-B, HLA-C, HLA-DRB1, and HLA-DQB1, were tested for patient genotyping. Briefly, 2 ml of human blood samples were collected in EDTA anticoagulant tube and DNA samples were extracted. The resulting genomic DNAs were sent to CapitalBio Technology (Beijing, China) for PCR amplification. The PCR condition used is: heating at 96 °C for 3 min, 35 cycles of denaturation at 96 °C for 25 s, annealing at 62 °C for 45 s, and extension at 72 °C for 45 s, and then a final extension at 72 °C for 5 min. The remaining primers in the PCR products were then digested by incubation with *Exo*I at 37 °C for 15 min. Afterward, *Exo*I was inactivated by incubation at 80 °C for 20 min. PCR products were then purified and sequenced using high-resolution ABI 3730XL sequencer (Applied Biosystems, Tampa, CA). Sequencing results were analyzed using ATF genotyping software (Conexio Genomics, Fremantle, Australia).

### Isolation of PBMCs, CD4^+^, CD8^+^ T cells

We used LymphoPrep™ (Axis-shield, Dundee, Scotland, UK) to isolate PBMCs as described previously [22]. CD4^+^ and CD8^+^ T cells were isolated from PBMCs using magnetic microbeads according to the manufacturer’s instructions (Miltenyi Biotec, Bergisch Gladbach, Germany, Cat. No.: 130-045-101 and 130-045-201). Firstly, PBMCs were aliquoted into 2 eppendorf tubes and incubated with either CD4 MicroBeads or CD8 MicroBeads for 15 min at 4 °C in the dark to magnetically label the CD4^+^ T cells and CD8^+^ T cells, respectively. Then, the cell suspensions were loaded onto a MACS column and placed in the magnetic field of a MACS separator (Miltenyi Biotec, Bergisch Gladbach, Germany). The magnetically-labeled CD4^+^ and CD8^+^ T cells were retained within the column while the unlabeled cells run through the column. The magnetically-retained T cells in the column were then eluted as the positively-selected cell fraction.

### *TCR*β primer design and validation

Human *TCR*β sequences (GenBank accession No. NG_001333) were downloaded from the international IMGT database [28]. We designed multiple primers for the *TCR*β sequences and validated the primers using the similar method as described previously [22]. Primer sequences are listed in Table S3.

### Sequencing library preparation

To prepare the *TCR*β sequencing library, we performed multiplex PCR to amplify the *CDR3* region of the *TCR*β gene using the primer set with 30 forward primers and 13 reverse primers as described in Table S3. Genomic DNA isolated from CD4 or CD8 T cell subsets was used as template for PCR amplification. PCR products were purified using AMPure XP beads (Beckman Coulter, Indianapolis, IN, Cat. No. A63881) to remove PCR primers and other impurities. Sequencing indices and adaptors were added to the immune library at the second round of PCR. The PCR conditions for adding indices were heating at 98 °C for 1 min, followed by 25 cycles of denaturation at 98 °C for 20 s, annealing at 65 °C for 30 s, and extension at 72 °C for 30 s, with a final extension at 72 °C for 7 min. PCR products were then subjected to gel electrophoresis for separation and the corresponding bands were excised for DNA purification by using QIAquick Gel Extraction Kit (Qiagen, Hilden, Germany). The resulting DNA was used as the library for sequencing on the Illumina HiSeq 2000/Miseq sequencing platform (Illumina, San Diego, CA).

### Data analysis

A total of 18,976,912 pair-end reads were generated by the Illumina sequencing platform (Table S4). We used FLASH software [48] to merge overlapping paired-end reads and obtained 16,376,727 raw reads. IgBLAST was used to perform the alignment of the merged reads to V, D, and J gene in germline references [49]. The reference sequences of V, D, and J gene in germline were obtained from IMGT. Reads with low alignment identity (<70%) to germline references were excluded. After read filtering, 11,114,426 reads were retained for further analysis. The starting and ending positions of the CDR3 region, reading frame, and productivity were identified according to the definition of IMGT [28].

We followed previous studies and defined that TCR clones with a frequency ⩾1% were considered to be HECs [24], [50]. Normalized Shannon entropy was used as an index to evaluate the diversity of the TCR repertoire:

(1) $$\text{H}(\text{X})=-\overset{n}{\underset{i=1}{\sum }}\frac{p(x_{i})\text{log}(p(x_{i}))}{\text{log}(n)}$$

where *p*(*x_i_*) is the frequency of TCR clone, *n* represents the total number of TCR clones, and *x_i_* indicates a particular TCR clone. Unpaired 2-tailed *t*-test is applied to calculate the significance level of differences of Shannon entropy among T1D patients, T2D patients, and healthy controls.

We developed an online web server iRAP for immune repertoire analysis, which is freely available for public use and can be accessed at http://www.sustc-genome.org.cn/irap2/index.php.

## Authors’ contributions

ZL, JH, and LX designed the project. ZL, HZ, MZ, YX, ZW, and XS performed the experiments. YT performed all the bioinformatics analysis of data. YT, ZL, MWD, and JH wrote the manuscript. All authors read and approved the final manuscript.

## Competing interests

The authors have no conflicts of interest to declare.

## Supplementary material

Supplementary Figure S1 Clone distribution in diabetic patients and nondiabetic controls **A.** Clone distribution in normalized sample size. We randomly sampled sequencing reads into the same size (50,000 reads) to reduce the bias induced from sequencing depth. Each data point represents a CDR3 clone. Y axis shows the frequency of each clone observed in different samples, expressed as the number of a specific TCR clone against the total number of CDR3 sequences in respective samples (%). HECs (frequency ⩾1%) are shown in red and LECs (frequency <1%) are shown in black. **B**. Shannon-entropy, HEC count, and HEC ratio for clonal distribution in the normalized samples. **C.** Subsampling at various depths. We recalculated total CDR3 clones into the same sequencing depth by random sampling method. The T1D, T2D, and control samples display similar trend, indicating that the subsampling approach is robust. HEC, highly-expanded clone; T1D, type 1 diabetes mellitus; T2D, type 2 diabetes mellitus. Statistical analysis was performed using student *t*-test (∗∗*P* < 0.05; ∗*P* < 0.01).

Supplementary Figure S2 The VJ combination usage patterns The VJ combination of individual samples is analyzed in the 2-dimensional plot. The X and Y axes list all possible V gene and J usages, respectively, while each point in the 2-dimensional space represents a unique VJ combination. The size of the sphere at each point represents the number of reads that match to a particular VJ combination.

Supplementary Figure S3 *TRBV* gene usage patterns in CD4 and CD8 T cells The white, gray and black bars represent the control, T2D and T1D samples. The bar charts show the percentage of each *TRBV* used in CD4^+^ (**A**) and CD8^+^ T (**C**) cells from each group on average. To find out *TRBV* genes that are significantly highly expressed in CD4^+^ (**B**) and CD8^+^ T (**D**) cells from T1D samples, we calculated the *P* value by comparing control to T1D, and comparing T2D to T1D, using student *t*-test. The green dashed horizontal lines represent the significance level (*P* < 0.01). T1D, type 1 diabetes mellitus; T2D, type 2 diabetes mellitus.

Supplementary Figure S4 VJ recombination clustering The T1D patients are grouped into a cluster, except sample P6. TRBV9–TRBJ2-7 combination is significantly more used in CD4^+^ cells of T1D patients (*P* = 0.031; **A**), while TRBV15–TRBJ2-5 is significantly more used in CD8^+^ cells of T1D patients (*P* = 0.026; **B**). Statistical analysis was performed using student *t*-test. The frequency of combinatorial VJ usage is color-coded in the figure. T1D, type 1 diabetes mellitus.

Supplementary Figure S5 Shared clones between T1D, T2D and control samples The complete list of shared HECs is shown in the heatmap. Orange and green represent HECs (⩾1% of total reads) and LECs (<1% of total reads), respectively, while gray represents sequences not found in the sample. HEC, highly-expanded clone; LEC, lowly-expanded clone; T1D, type 1 diabetes mellitus; T2D, type 2 diabetes mellitus.

Supplementary Table S1 High-resolution HLA genotyping for 6 T1D patients

Supplementary Table S2 Clinical information for subjects included

Supplementary Table S3 Primer sequences for amplification of human TCR CDR3

Supplementary Table S4 Summary of sequencing data

## References

[1] Katz J.D., Benoist C., Mathis D. (1995). T helper cell subsets in insulin-dependent diabetes. Science.

[2] Roep B.O. (2003). The role of T-cells in the pathogenesis of Type 1 diabetes: from cause to cure. Diabetologia.

[3] Cnop M., Welsh N., Jonas JC, Jörns A., Lenzen S., Eizirik D.L. (2005). Mechanisms of pancreatic β-cell death in Type 1 and Type 2 diabetes many differences, few similarities. Diabetes.

[4] Somoza N., Vargas F., Roura-Mir C., Vives-Pi M., Fernández-Figueras M.T., Ariza A. (1994). Pancreas in recent onset insulin-dependent diabetes mellitus. Changes in HLA, adhesion molecules and autoantigens, restricted T cell receptor V beta usage, and cytokine profile. J Immunol.

[5] Kelemen K. (2004). The role of T cells in beta cell damage in NOD mice and humans. Adv Exp Med Biol.

[6] Haskins K. (2005). Pathogenic T-cell clones in autoimmune diabetes: more lessons from the NOD mouse. Adv Immunol.

[7] Planas R., Carrillo J., Sanchez A., de Villa M.C., Nunez F., Verdaguer J. (2010). Gene expression profiles for the human pancreas and purified islets in type 1 diabetes: new findings at clinical onset and in long-standing diabetes. Clin Exp Immunol.

[8] Miyazaki A., Hanafusa T., Yamada K., Miyagawa J., Fujino-Kurihara H., Nakajima H. (1985). Predominance of T lymphocytes in pancreatic islets and spleen of pre-diabetic non-obese diabetic (NOD) mice: a longitudinal study. Clin Exp Immunol.

[9] Makino S. (1998). Genetic analysis of IDDM in NOD mice. Exp Anim.

[10] Wicker L.S., Miller B.J., Mullen Y. (1986). Transfer of autoimmune diabetes mellitus with splenocytes from nonobese diabetic (NOD) mice. Diabetes.

[11] Bendelac A., Carnaud C., Boitard C., Bach J.F. (1987). Syngeneic transfer of autoimmune diabetes from diabetic NOD mice to healthy neonates. Requirement for both L3T4+ and Lyt-2+ T cells. J Exp Med.

[12] Roberts S.A., Barbour G., Matarrese M.R., Mason D.L., Leiter E.H., Haskins K. (2003). Adoptive transfer of islet antigen-autoreactive T cell clones to transgenic NOD.Ea(d)mice induces diabetes indicating a lack of I-E mediated protection against activated effector T cells. J Autoimmun.

[13] Bottazzo G.F., Dean B.M., McNally J.M., MacKay E.H., Swift P.G., Gamble D.R. (1985). *In situ* characterization of autoimmune phenomena and expression of HLA molecules in the pancreas in diabetic insulitis. N Engl J Med.

[14] Eizirik D.L., Mandrup-Poulsen T. (2001). A choice of death-the signal-transduction of immune-mediated beta-cell apoptosis. Diabetologia.

[15] Wagner U.G., Koetz K., Weyand C.M., Goronzy J.J. (1998). Perturbation of the T cell repertoire in rheumatoid arthritis. Proc Natl Acad Sci U S A.

[16] Jiang H., Zhang S.I., Pernis B. (1992). Role of CD8^+^ T cells in murine experimental allergic encephalomyelitis. Science.

[17] Zhang X.M., Heber-Katz E. (1992). T cell receptor sequences from encephalitogenic T cells in adult Lewis rats suggest an early ontogenic origin. J Immunol.

[18] Goronzy J.J., Qi Q., Olshen R.A., Weyand C.M. (2015). High-throughput sequencing insights into T-cell receptor repertoire diversity in aging. Genome Med.

[19] Simone E., Daniel D., Schloot N., Gottlieb P., Babu S., Kawasaki E. (1997). T cell receptor restriction of diabetogenic autoimmune NOD T cells. Proc Natl Acad Sci U S A.

[20] Li L., He Q., Garland A., Yi Z., Aybar L.T., Kepler T.B. (2009). β cell-specific CD4^+^ T cell clonotypes in peripheral blood and the pancreatic islets are distinct. J Immunol.

[21] Codina-Busqueta E., Scholz E., Munoz-Torres P.M., Roura-Mir C., Costa M., Xufre C. (2011). TCR bias of in vivo expanded T cells in pancreatic islets and spleen at the onset in human type 1 diabetes. J Immunol.

[22] Li Z., Liu G., Tong Y., Zhang M., Xu Y., Qin L. (2015). Comprehensive analysis of the T-cell receptor beta chain gene in rhesus monkey by high throughput sequencing. Sci Rep.

[23] Weinstein J.A., Jiang N., White R.A., Fisher D.S., Quake S.R. (2009). High-throughput sequencing of the zebrafish antibody repertoire. Science.

[24] Klarenbeek P.L., de Hair M.J., Doorenspleet M.E., van Schaik B.D., Esveldt R.E., van de Sande M.G. (2012). Inflamed target tissue provides a specific niche for highly expanded T-cell clones in early human autoimmune disease. Ann Rheum Dis.

[25] Jiang N., He J., Weinstein J.A., Penland L., Sasaki S., He X.S. (2013). Lineage structure of the human antibody repertoire in response to influenza vaccination. Sci Transl Med.

[26] Robins H. (2013). Immunosequencing: applications of immune repertoire deep sequencing. Curr Opin Immunol.

[27] Li S., Lefranc MP, Miles J.J., Alamyar E., Giudicelli V., Duroux P. (2013). IMGT/HighV QUEST paradigm for T cell receptor IMGT clonotype diversity and next generation repertoire immunoprofiling. Nat Commun.

[28] Giudicelli V., Chaume D., Lefranc M.P. (2005). IMGT/GENE-DB: a comprehensive database for human and mouse immunoglobulin and T cell receptor genes. Nucleic Acids Res.

[29] Lefranc M.P., Giudicelli V., Ginestoux C., Bodmer J., Müller W., Bontrop R. (1999). IMGT, the international ImMunoGeneTics database. Nucleic Acids Res.

[30] Chao A., Shen T.J. (2003). Nonparametric estimation of Shannon’s index of diversity when there are unseen species in sample. Environ Ecol Stat.

[31] Reijonen H., Mallone R., Heninger A.K., Laughlin E.M., Kochik S.A., Falk B. (2004). GAD65-specific CD4^+^ T-cells with high antigen avidity are prevalent in peripheral blood of patients with type 1 diabetes. Diabetes.

[32] Kent S.C., Chen Y., Bregoli L., Clemmings S.M., Kenyon N.S., Ricordi C. (2005). Expanded T cells from pancreatic lymph nodes of type 1 diabetic subjects recognize an insulin epitope. Nature.

[33] Marrero I., Hamm D.E., Davies J.D. (2013). High-throughput sequencing of islet-infiltrating memory CD4^+^ T cells reveals a similar pattern of TCR Vbeta usage in prediabetic and diabetic NOD mice. PLoS One.

[34] Woodsworth D.J., Castellarin M., Holt R.A. (2013). Sequence analysis of T-cell repertoires in health and disease. Genome Med.

[35] Robins H.S., Campregher P.V., Srivastava S.K., Wacher A., Turtle C.J., Kahsai O. (2009). Comprehensive assessment of T-cell receptor beta-chain diversity in alphabeta T cells. Blood.

[36] Roep B.O., Arden S.D., de Vries R.R., Hutton J.C. (1990). T-cell clones from a type-1 diabetes patient respond to insulin secretory granule proteins. Nature.

[37] Arif S., Tree T.I., Astill T.P., Tremble J.M., Bishop A.J., Dayan C.M. (2004). Autoreactive T cell responses show proinflammatory polarization in diabetes but a regulatory phenotype in health. J Clin Invest.

[38] Velthuis J.H., Unger W.W., Abreu J.R., Duinkerken G., Franken K., Peakman M. (2010). Simultaneous detection of circulating autoreactive CD8^+^ T-cells specific for different islet cell-associated epitopes using combinatorial MHC multimers. Diabetes.

[39] Lernmark A. (2001). Autoimmune diseases: are markers ready for prediction?. J Clin Invest.

[40] Filion M.C., Proulx C., Bradley A.J., Devine D.V., Sekaly R.P., Decary F. (1996). Presence in peripheral blood of healthy individuals of autoreactive T cells to a membrane antigen present on bone marrow-derived cells. Blood.

[41] van Belle T.L., Coppieters K.T., von Herrath M.G. (2011). Type 1 diabetes: etiology, immunology, and therapeutic strategies. Physiol Rev.

[42] Koretzky G.A. (2010). Multiple roles of CD4 and CD8 in T cell activation. J Immunol.

[43] Miceli M.C., Parnes J.R. (1991). The roles of CD4 and CD8 in T cell activation. Semin Immunol.

[44] Zhou Z., Reyes-Vargas E., Escobar H., Chang K.Y., Barker A.P., Rockwood A.L. (2016). Peptidomic analysis of type 1 diabetes associated HLA-DQ molecules and the impact of HLA-DM on peptide repertoire editing. Eur J Immunol.

[45] Singh S, Usha, Singh G, Agrawal NK, Singh RG, Kumar SB. Prevalence of autoantibodies and HLA DR, DQ in type 1 diabetes mellitus. J Clin Diagn Res 2016;10:EC09–13. 10.7860/JCDR/2016/18657.8163 PMC5020266 27630850

[46] Zhang J., Zhao L., Wang B., Gao J., Wang L., Li L. (2016). HLA-A*33-DR3 and A*33-DR9 haplotypes enhance the risk of type 1 diabetes in Han Chinese. J Diabetes Investig.

[47] Roden M. (2016). Diabetes mellitus: definition, classification and diagnosis. Wien Klin Wochenschr.

[48] Magoc T., Salzberg S.L. (2011). FLASH: fast length adjustment of short reads to improve genome assemblies. Bioinformatics.

[49] Ye J., Ma N., Madden T.L., Ostell J.M. (2013). IgBLAST: an immunoglobulin variable domain sequence analysis tool. Nucleic Acids Res.

[50] Kriangkum J., Motz S.N., Debes Marun C.S., Lafarge S.T., Gibson S.B., Venner C.P. (2013). Frequent occurrence of highly expanded but unrelated B-cell clones in patients with multiple myeloma. PLoS One.

