# Supplementary figures and images for: T Cell Repertoire Diversity Is Decreased in Type 1 Diabetes Patients
Source: Genomics Proteomics Bioinformatics. 2016 Dec 24;14(6):338–48. doi: 10.1016/j.gpb.2016.10.003 (PMC5200939; doi:10.1016/j.gpb.2016.10.003)

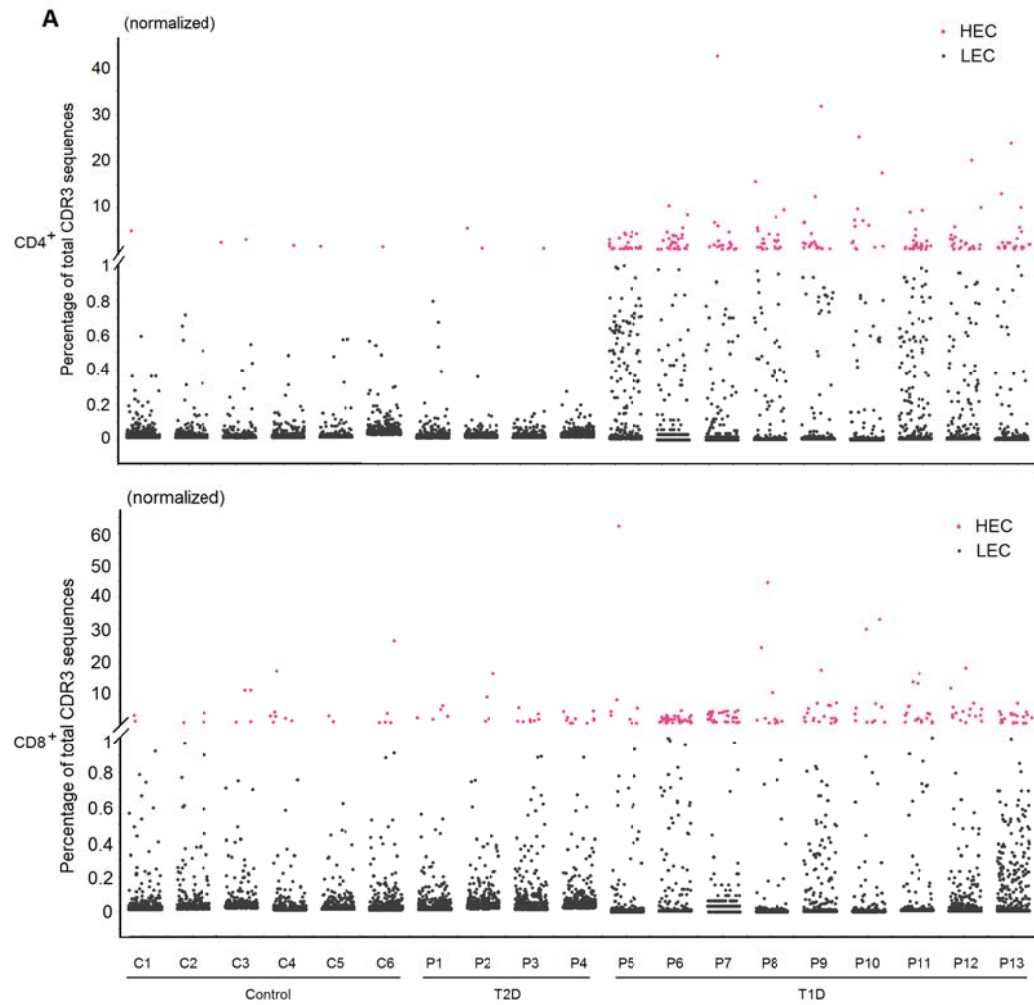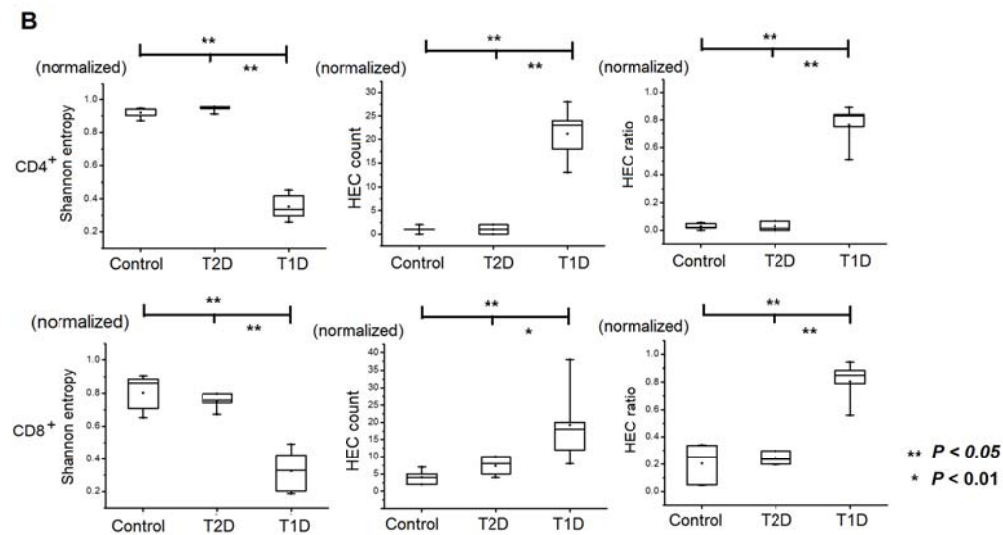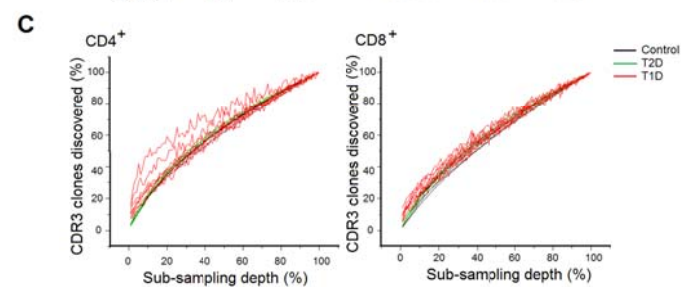

Supplement: Supplementary Figure S1 — Clone distribution in diabetic patients and nondiabetic controls A. Clone distribution in normalized sample size. We randomly sampled sequencing reads into the same size (50,000 reads) to reduce the bias induced from sequencing depth. Each data point represents a CDR3 clone. Y axis shows the frequency of each clone observed in different samples, expressed as the number of a specific TCR clone against the total number of CDR3 sequences in respective samples (%). HECs (frequency ⩾1%) are shown in red and LECs (frequency <1%) are shown in black. B. Shannon-entropy, HEC count, and HEC ratio for clonal distribution in the normalized samples. C. Subsampling at various depths. We recalculated total CDR3 clones into the same sequencing depth by random sampling method. The T1D, T2D, and control samples display similar trend, indicating that the subsampling approach is robust. HEC, highly-expanded clone; T1D, type 1 diabetes mellitus; T2D, type 2 diabetes mellitus. Statistical analysis was performed using student t-test (∗∗P < 0.05; ∗P < 0.01). [file mmc1.pdf]

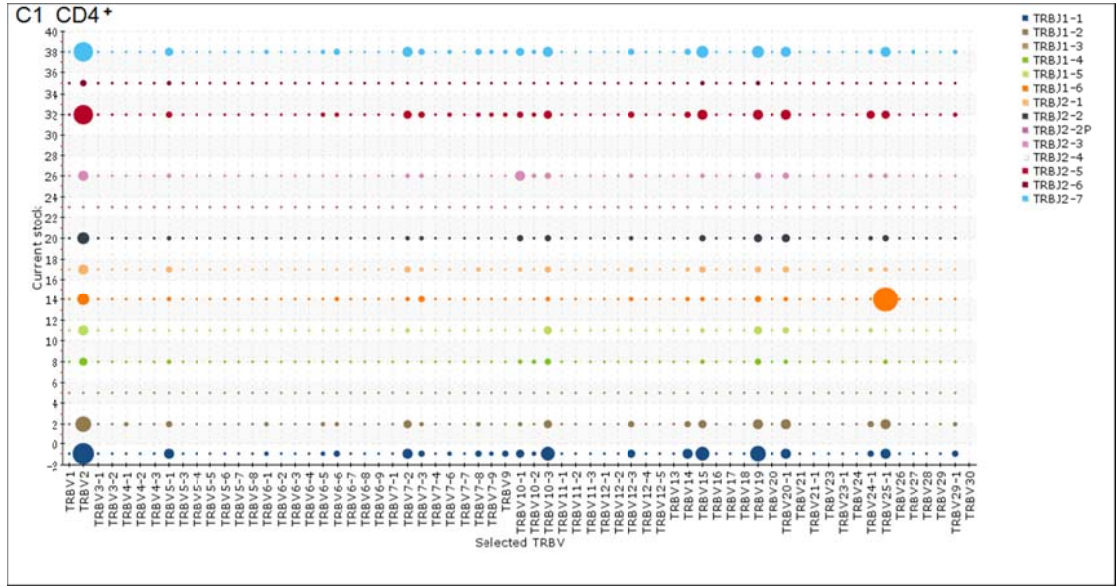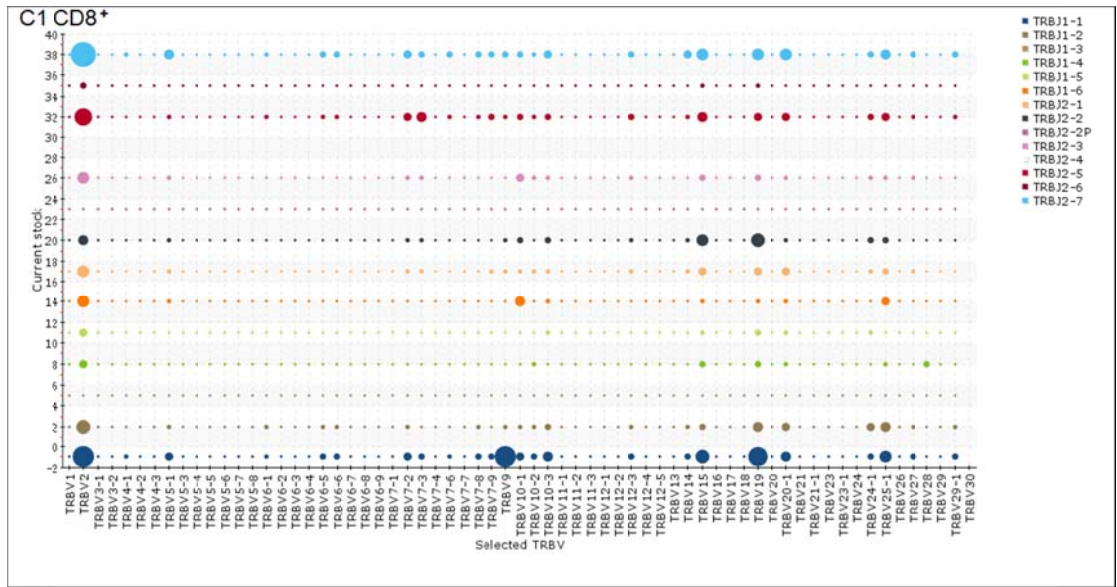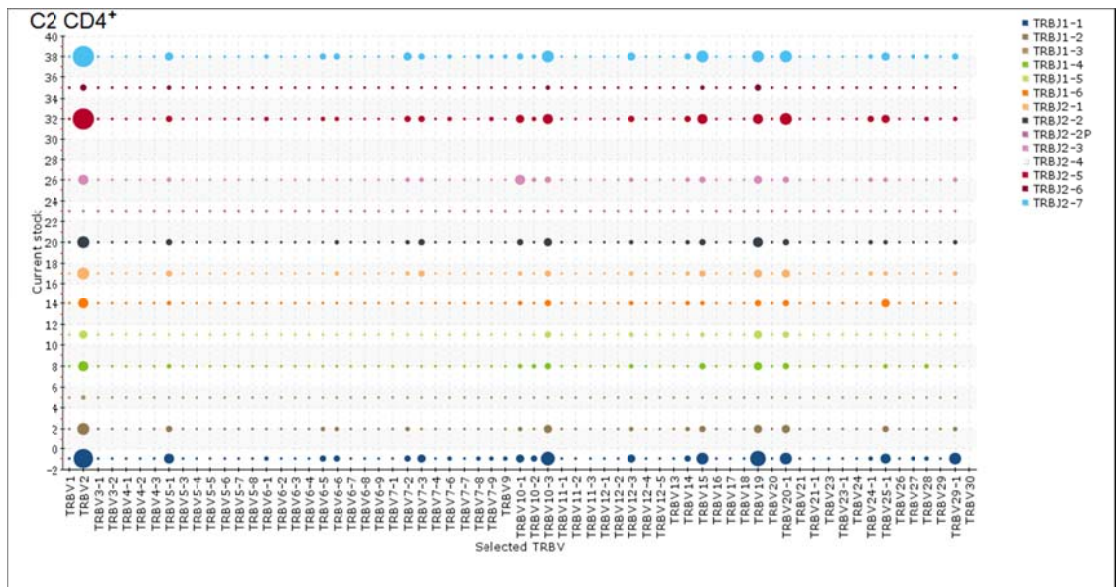

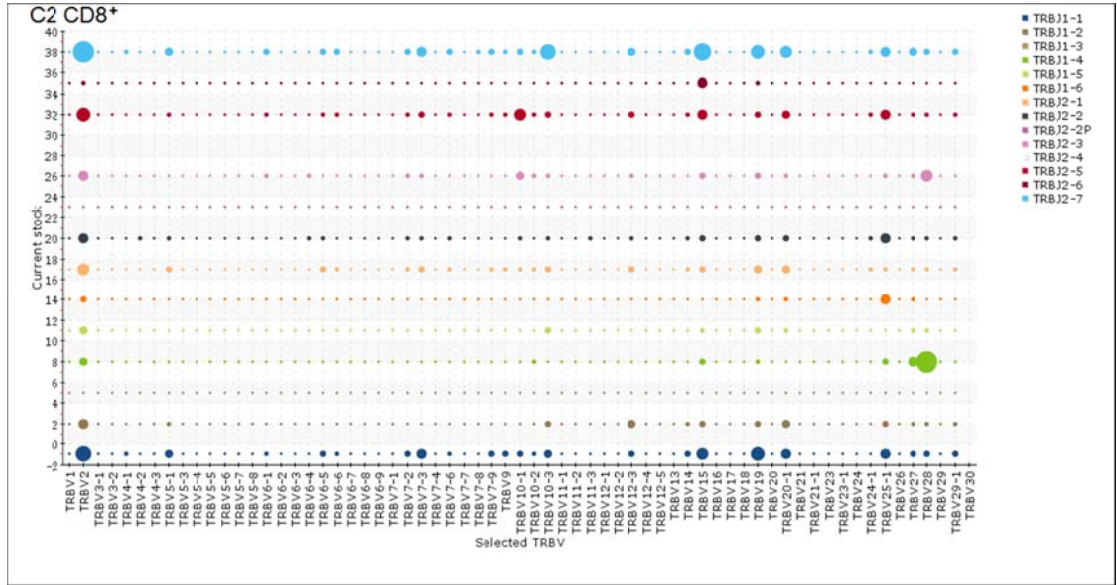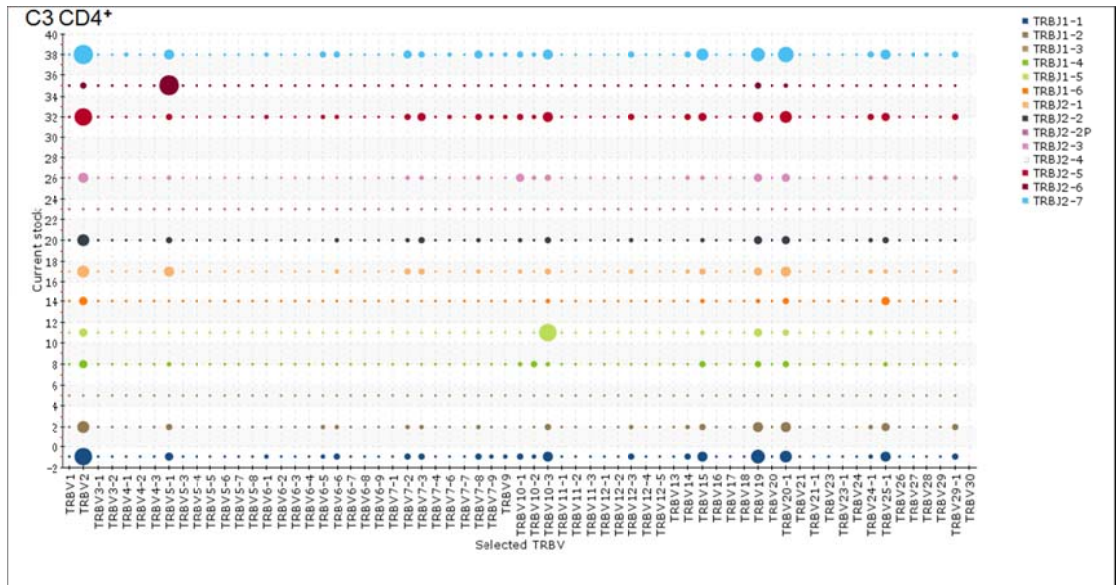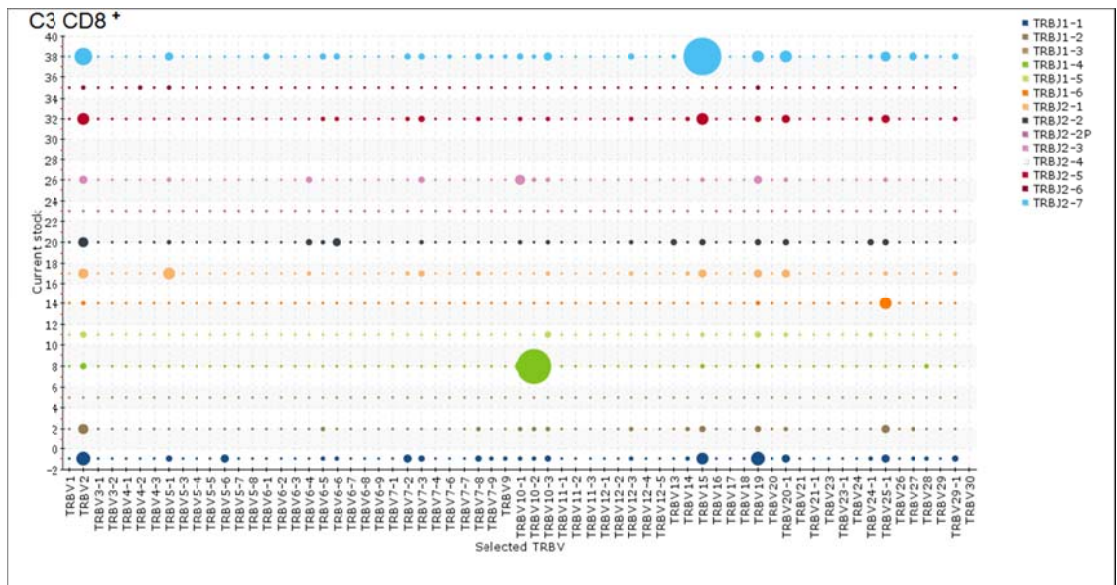

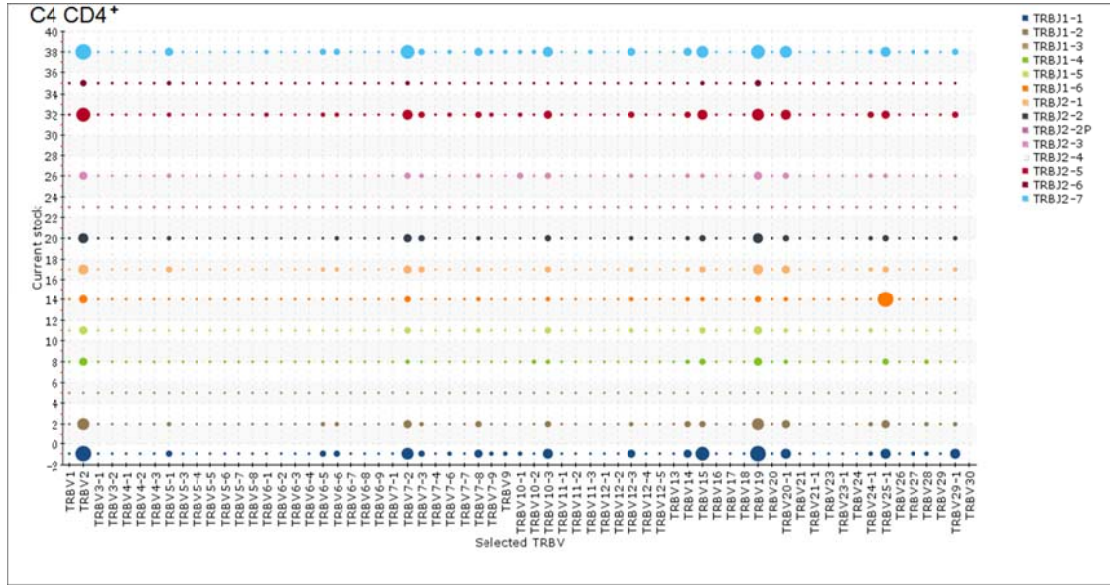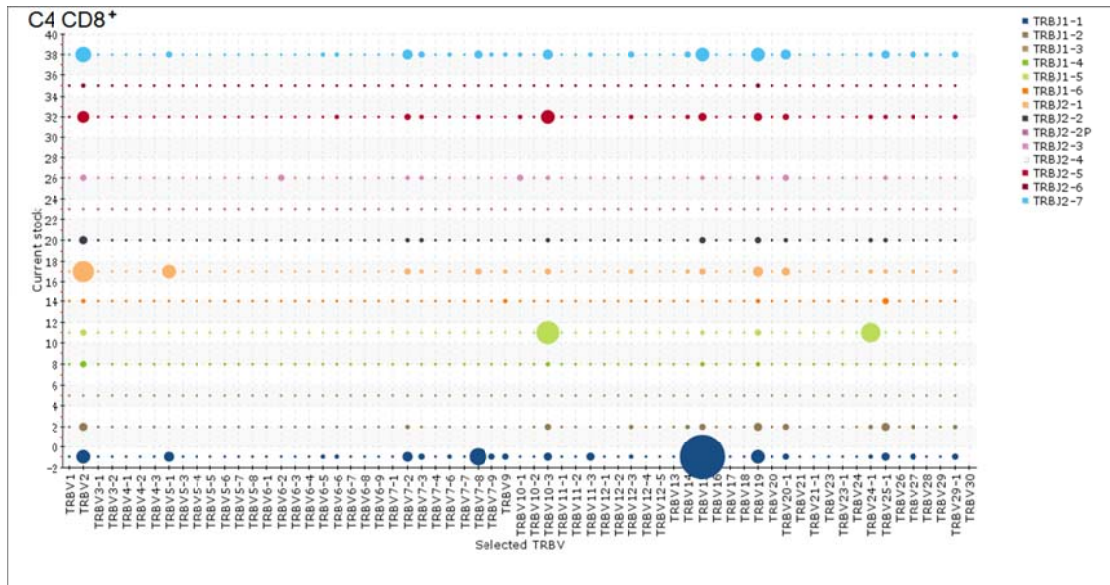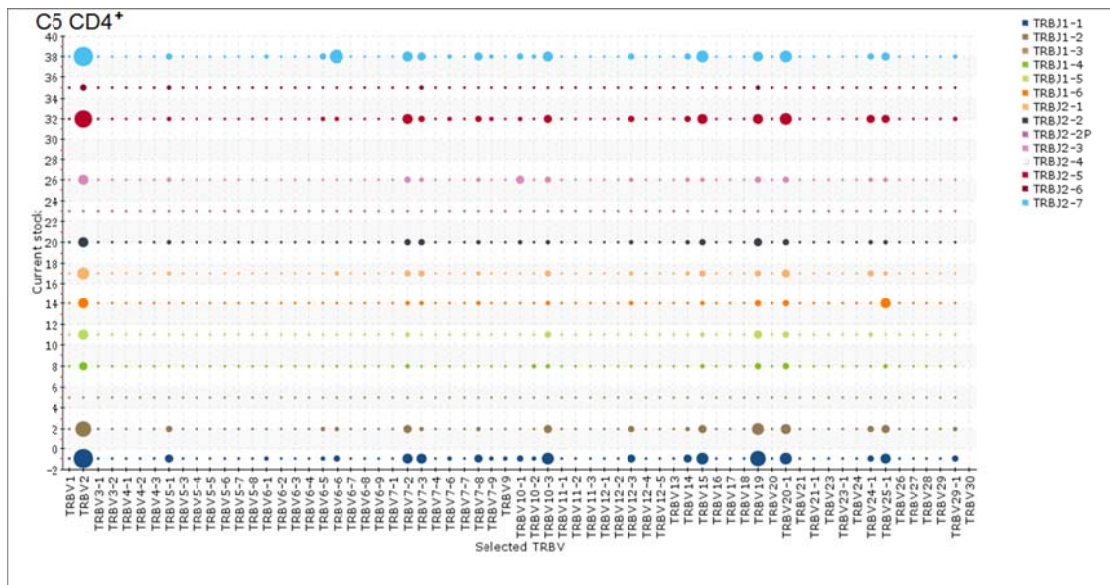

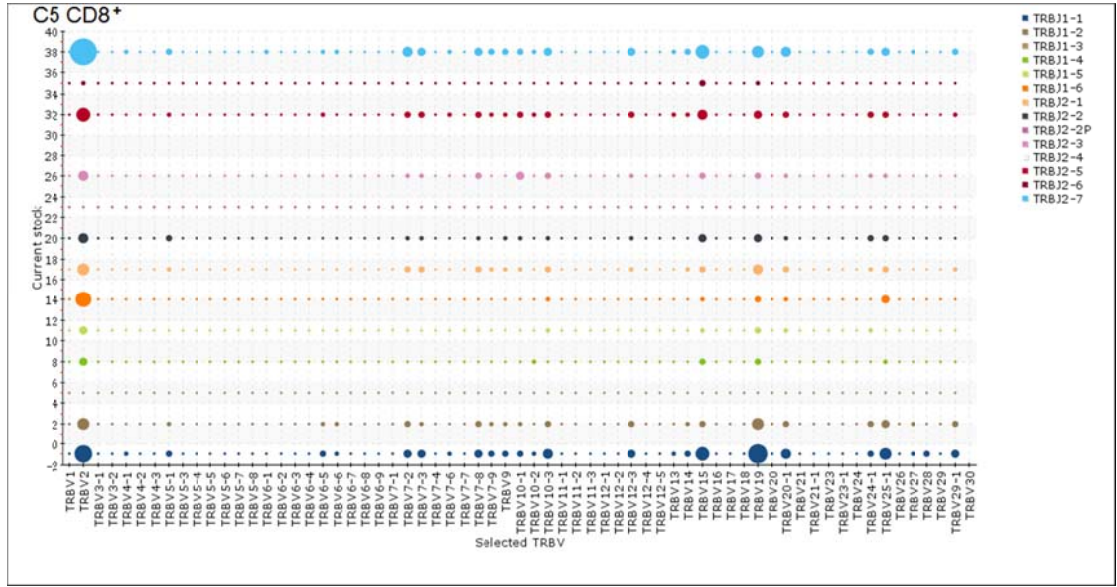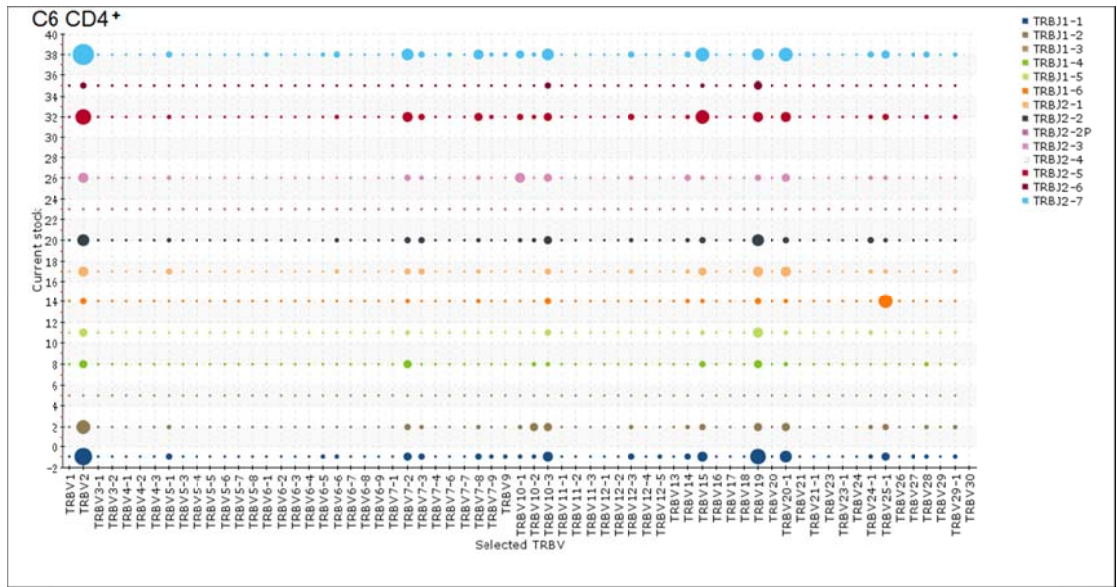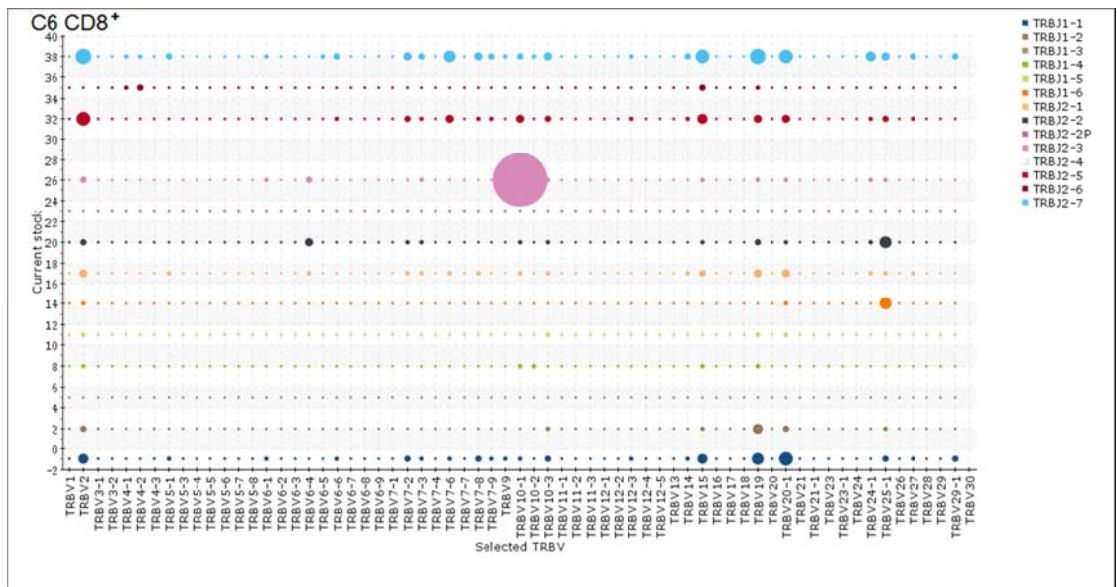

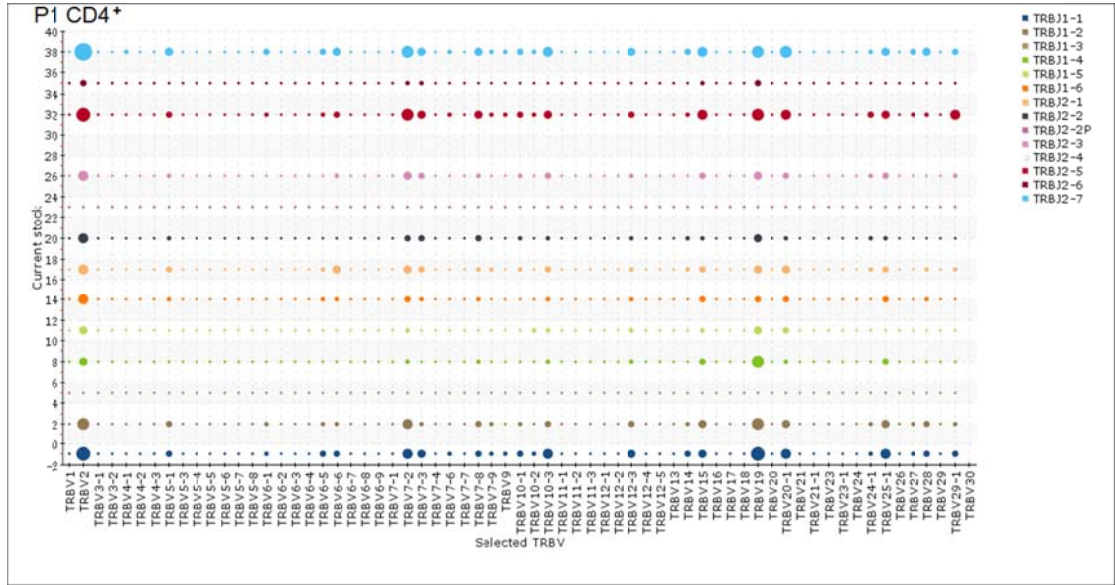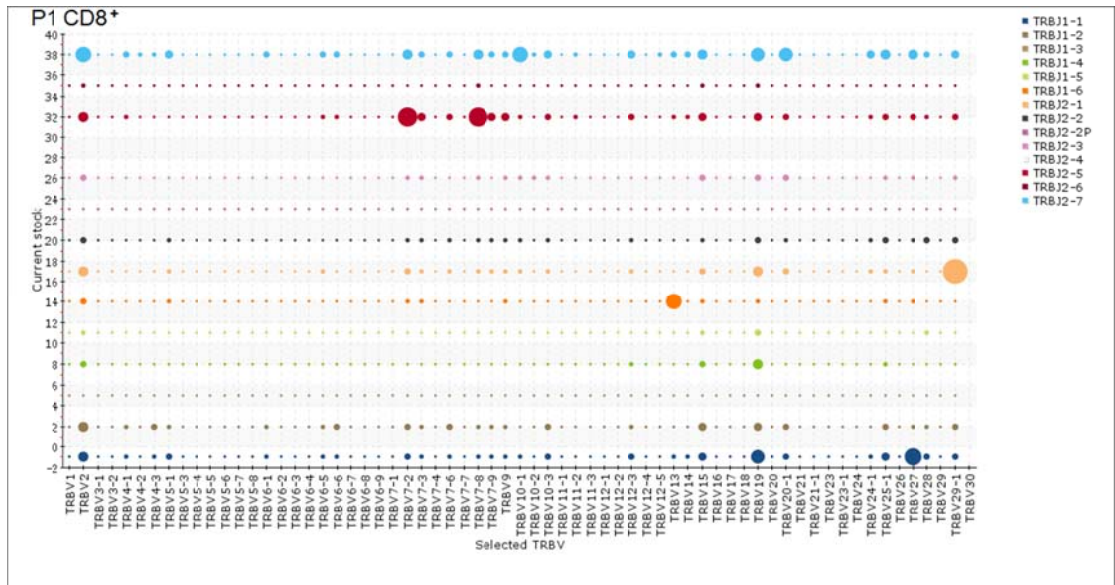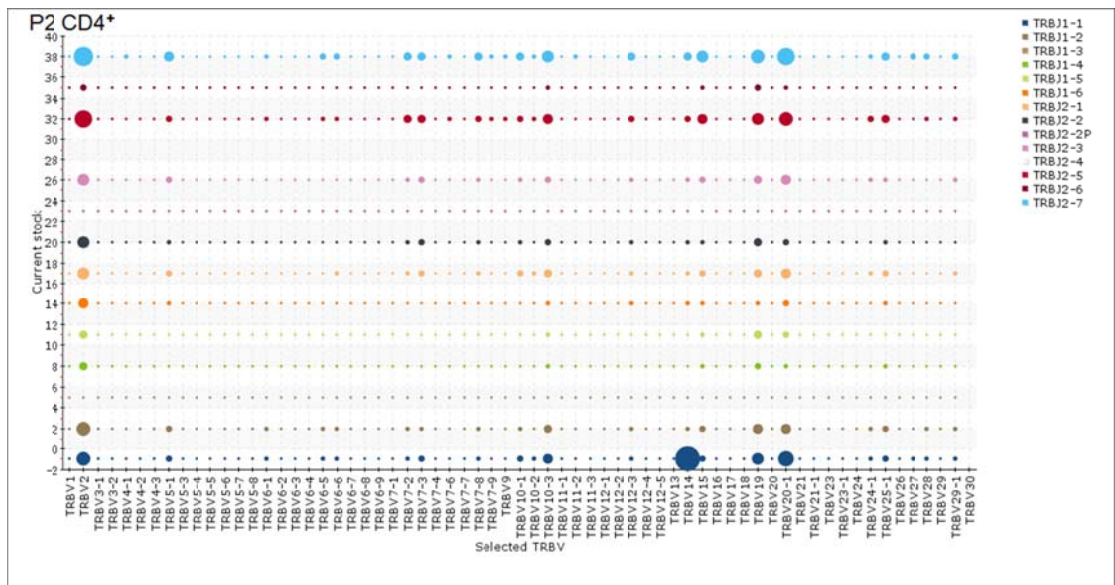

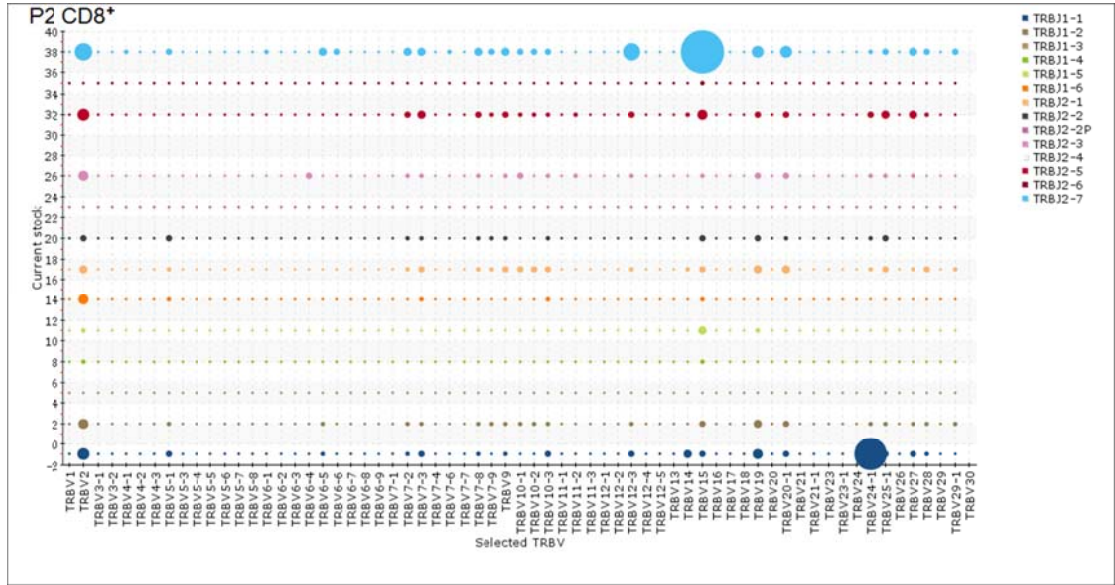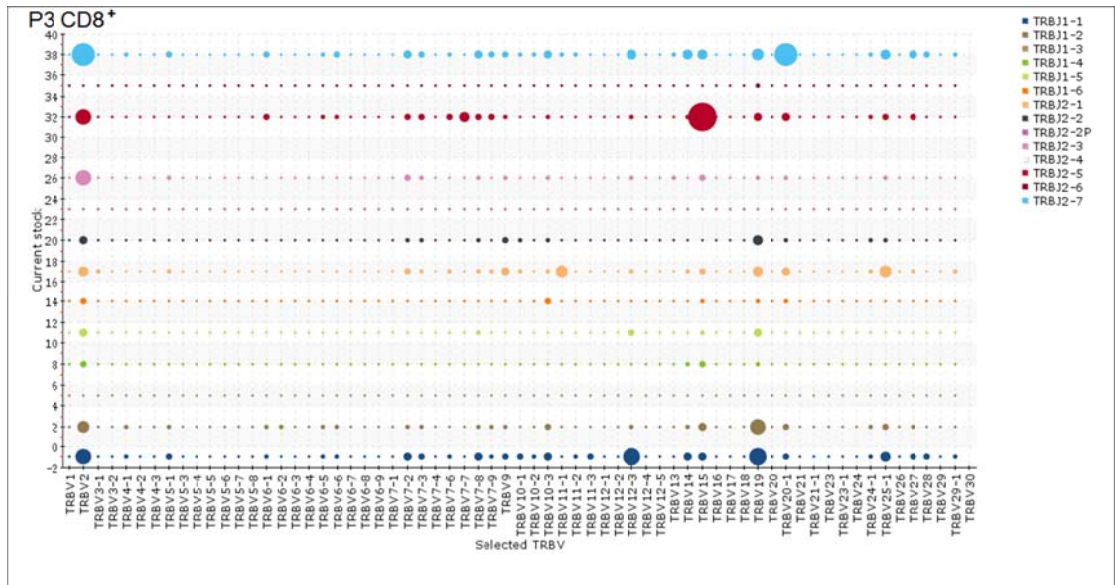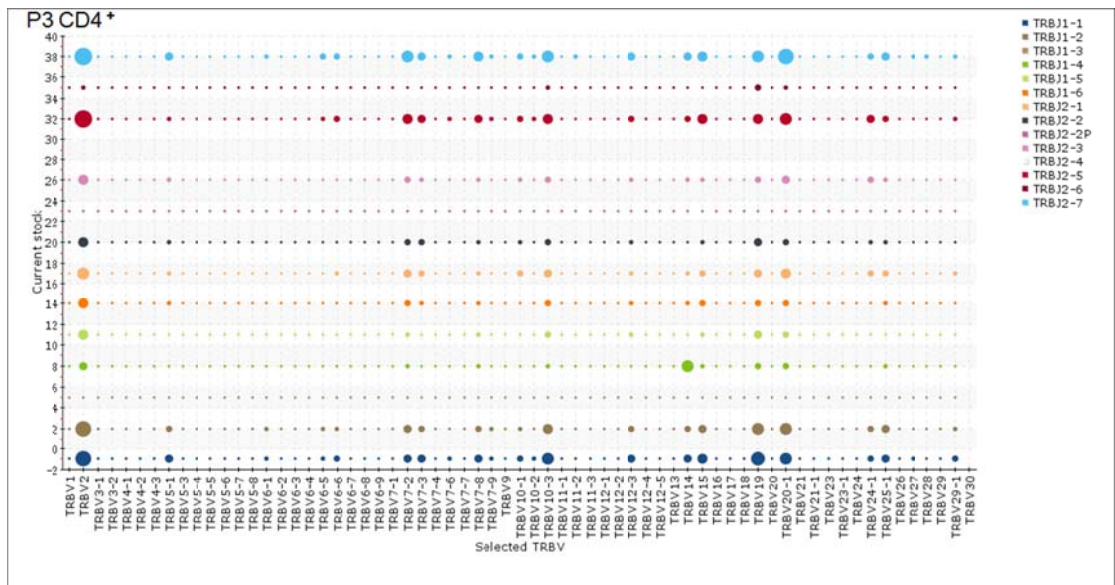

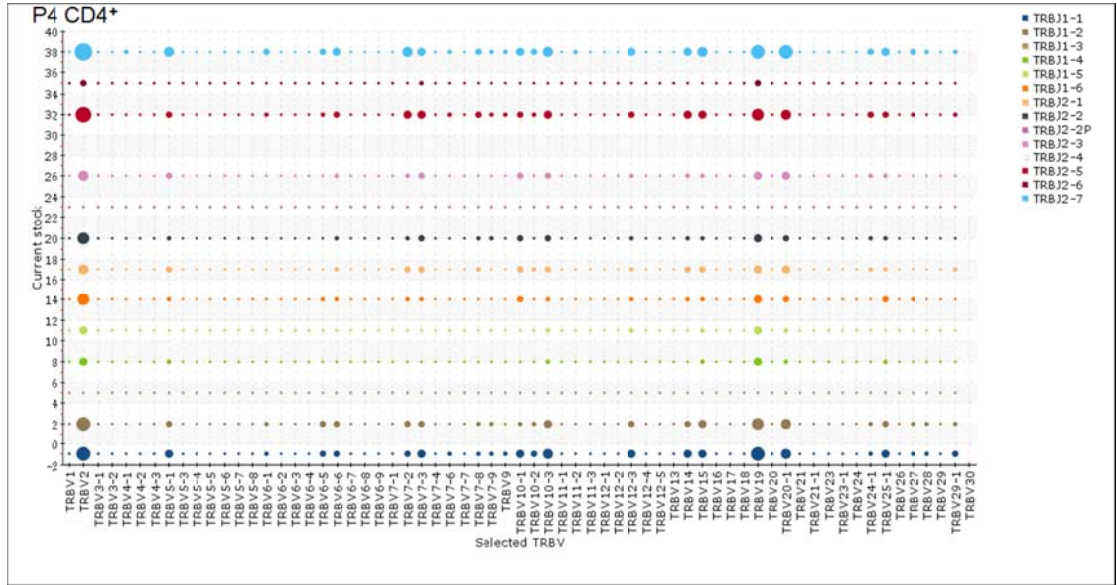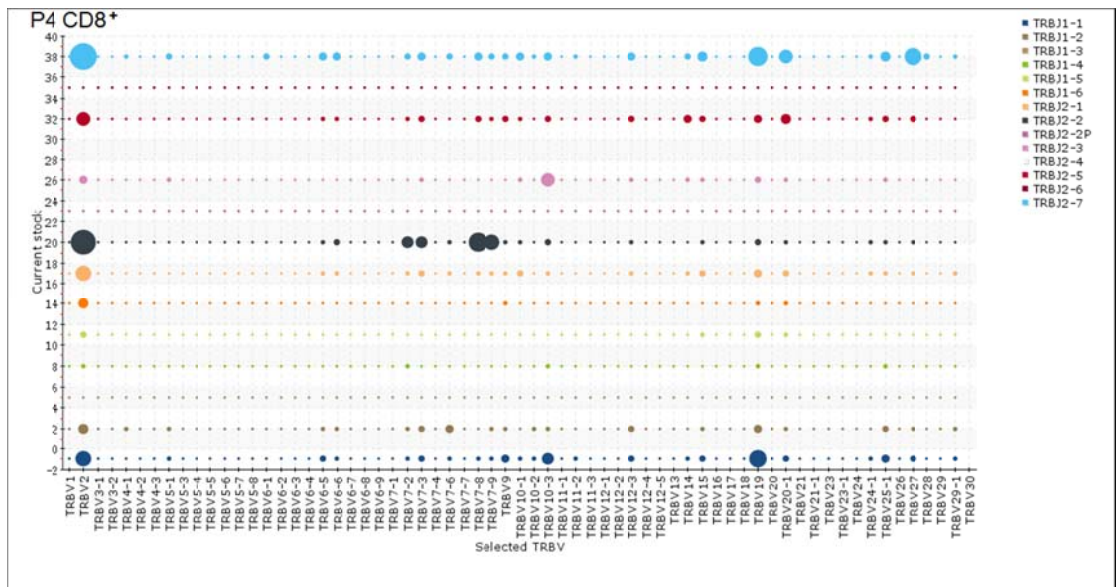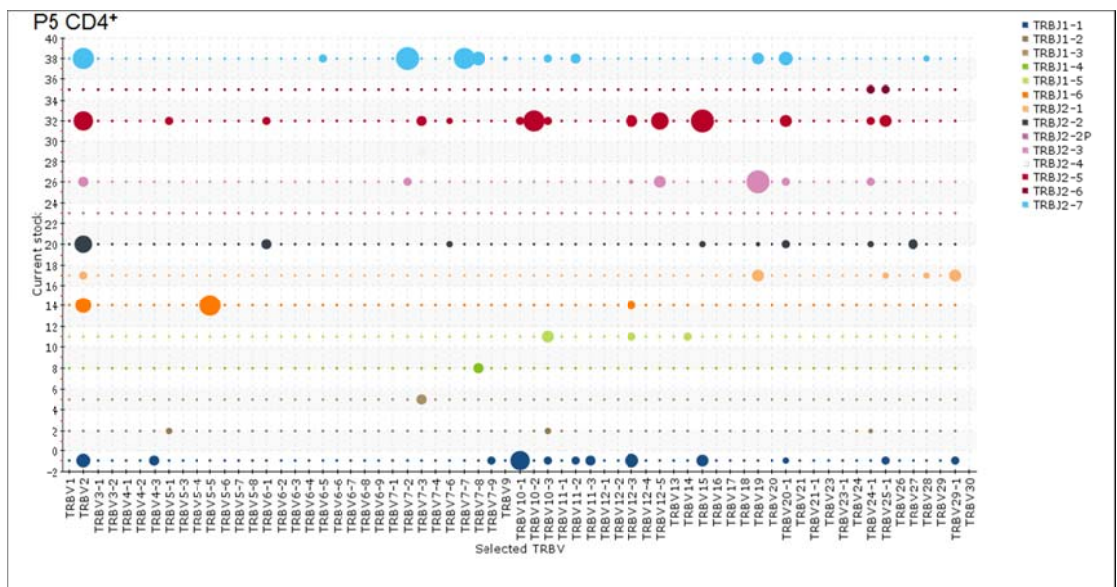

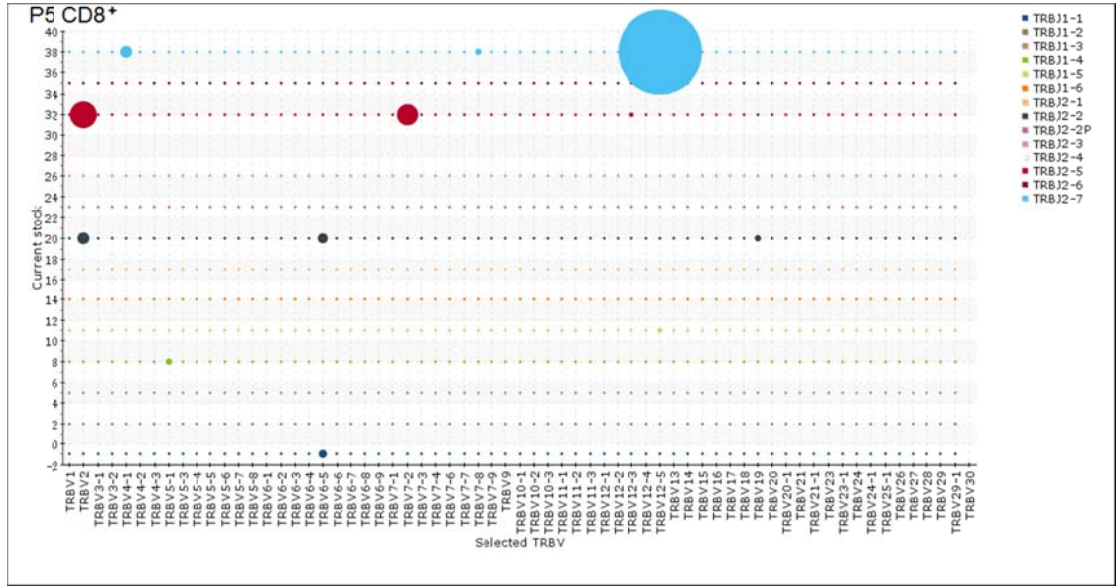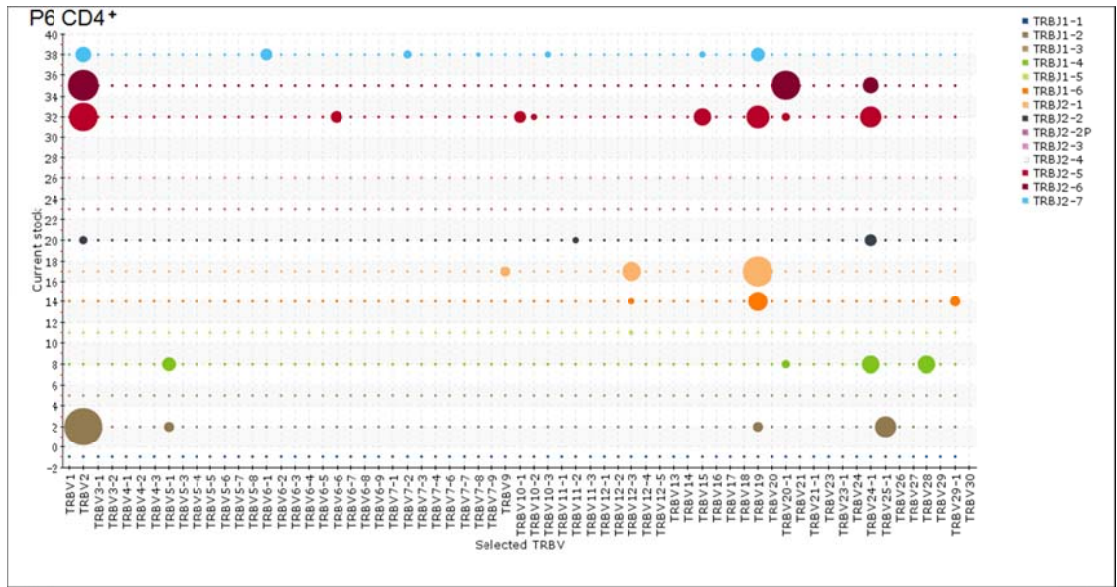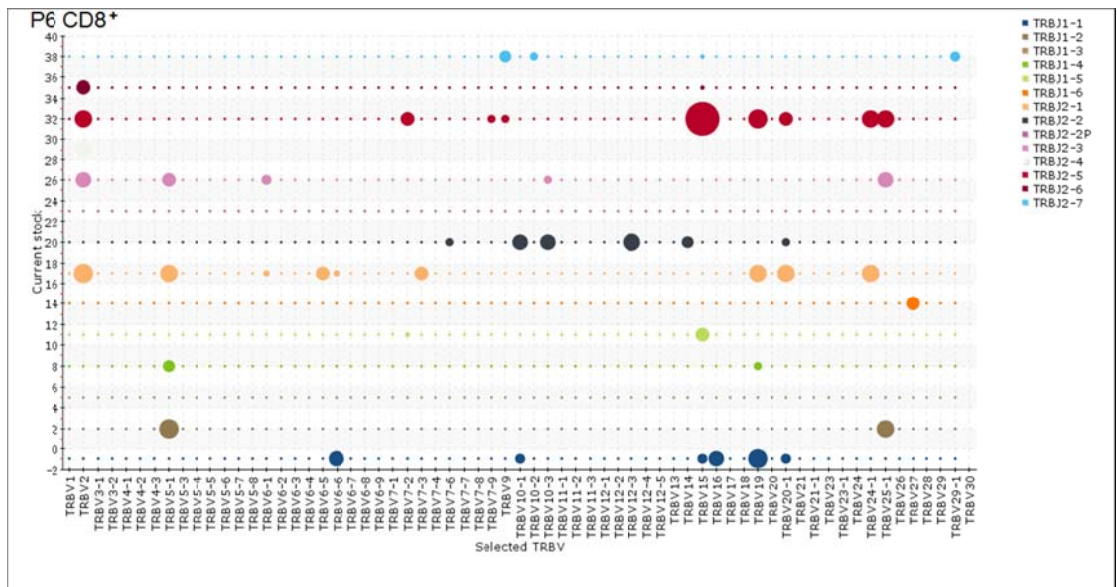

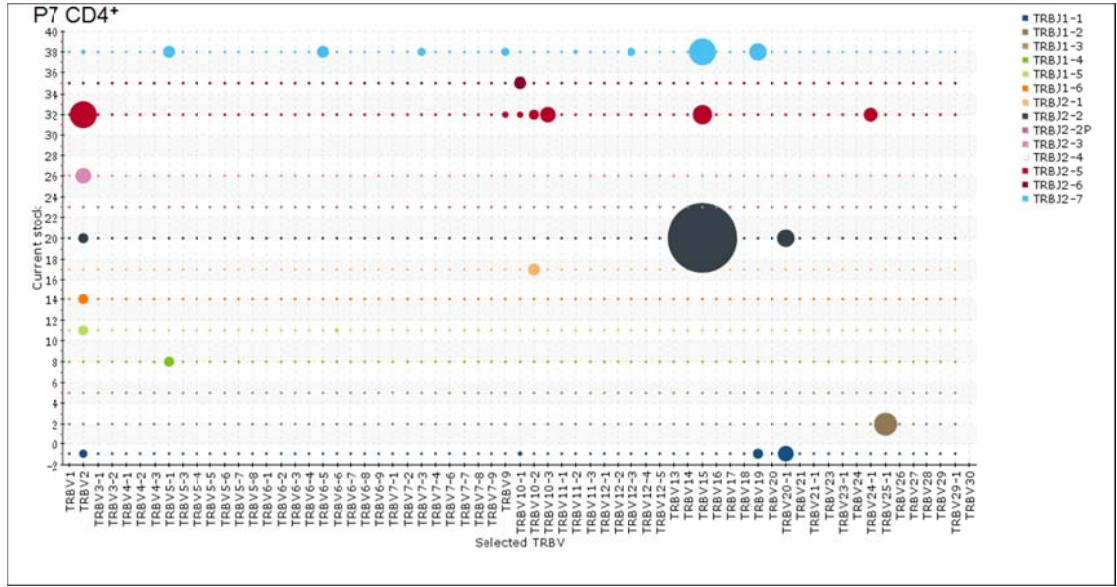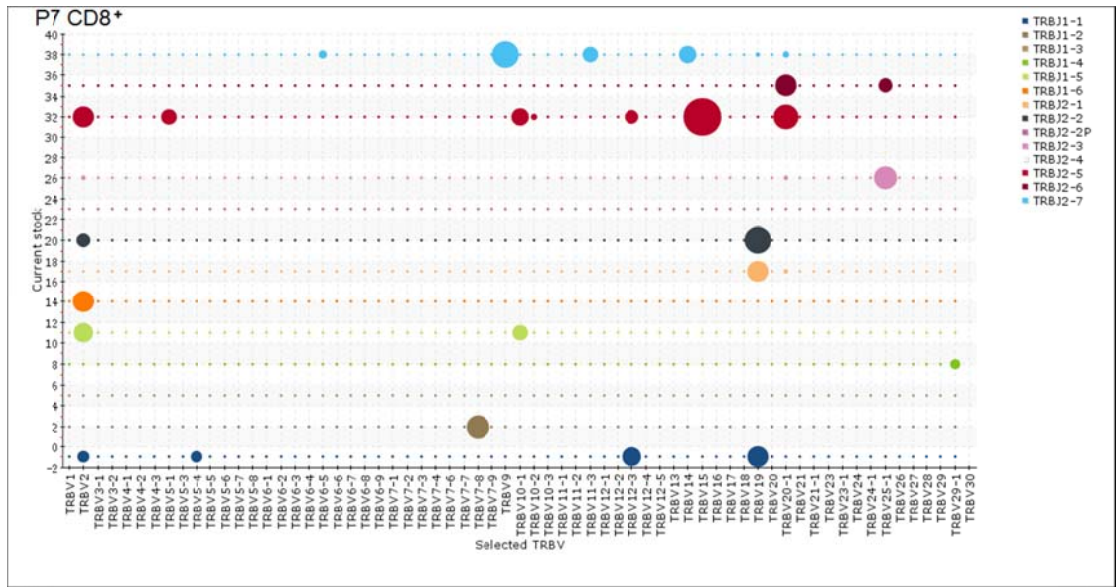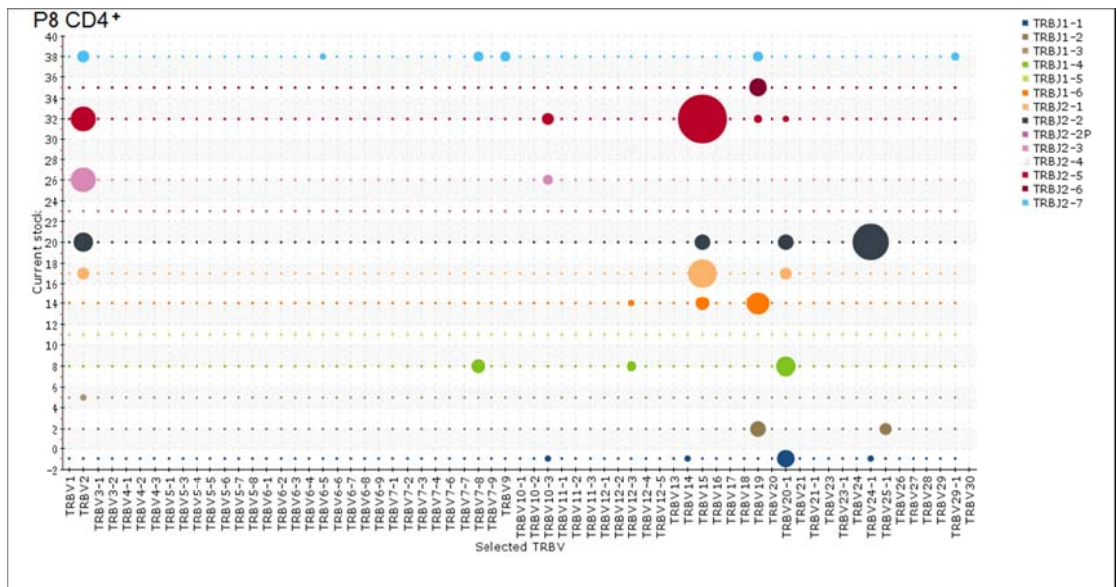



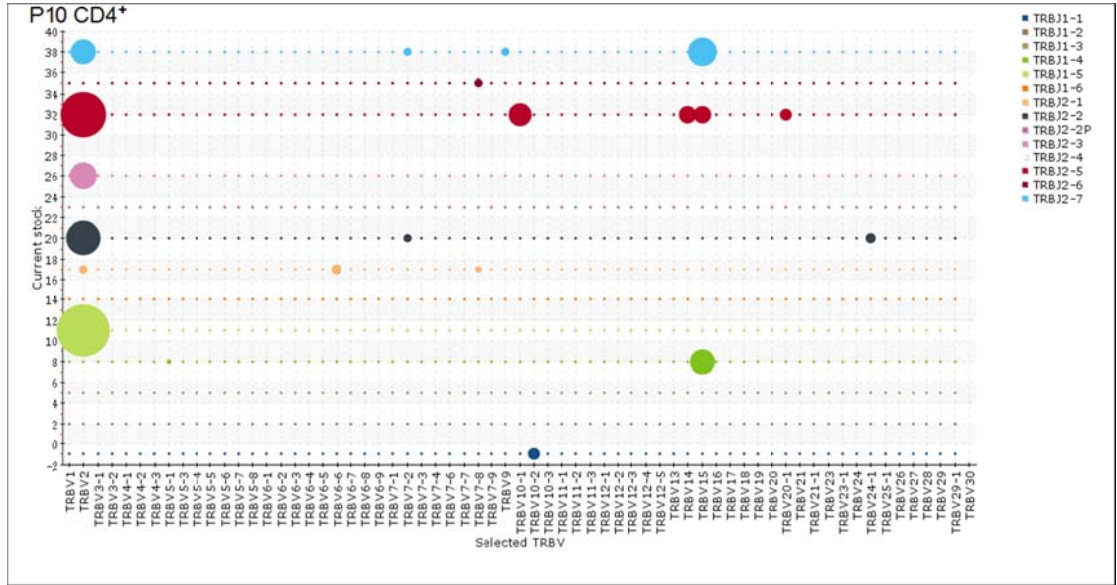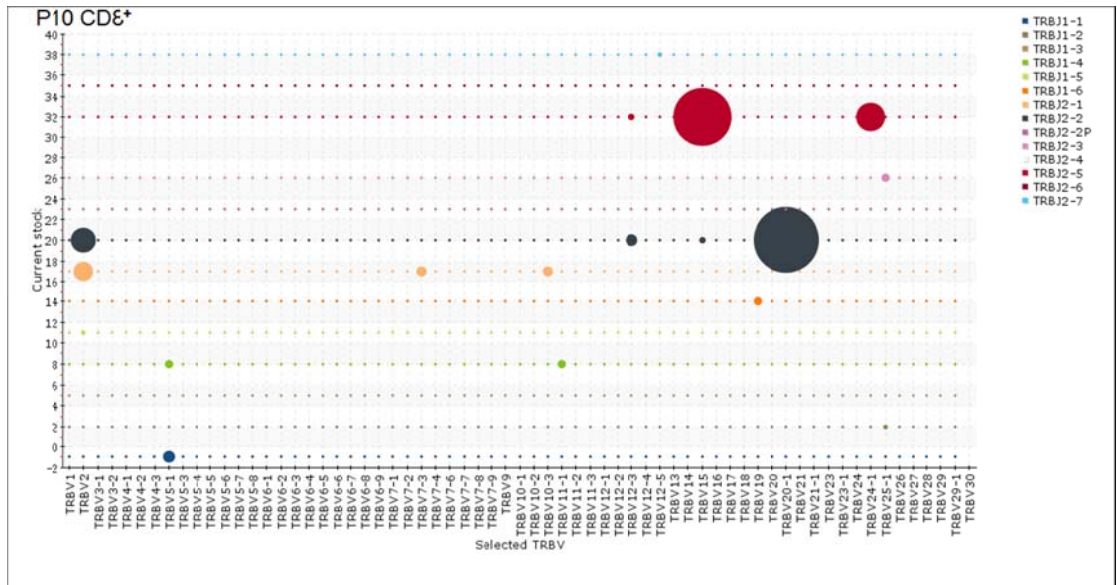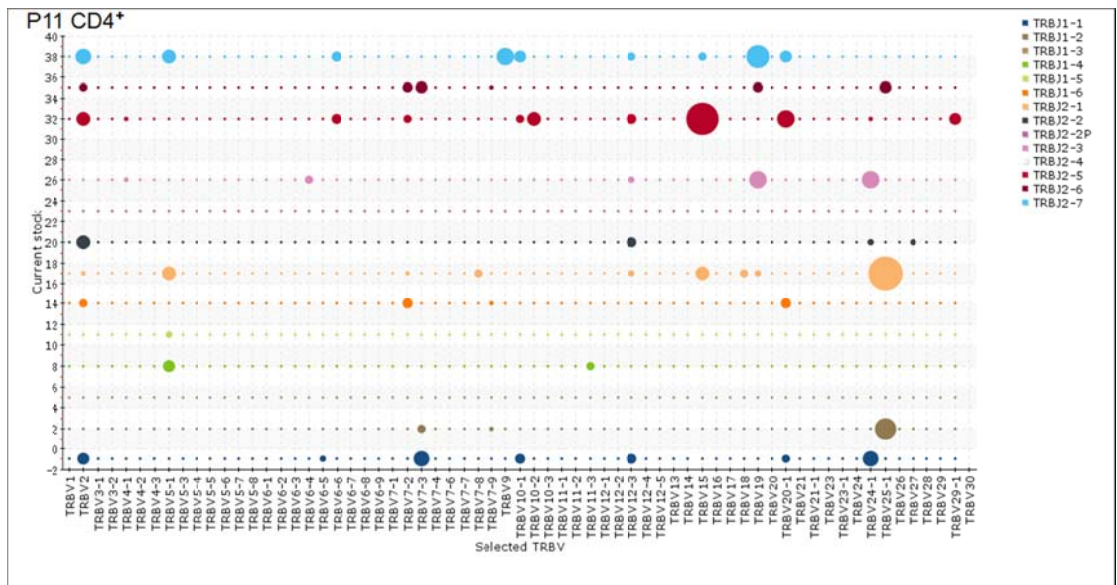

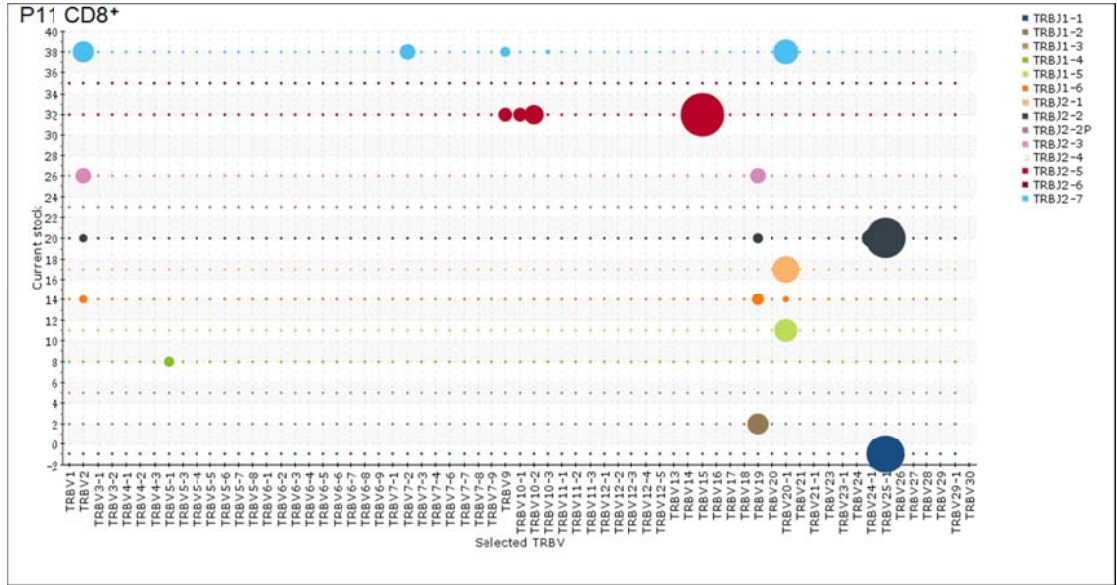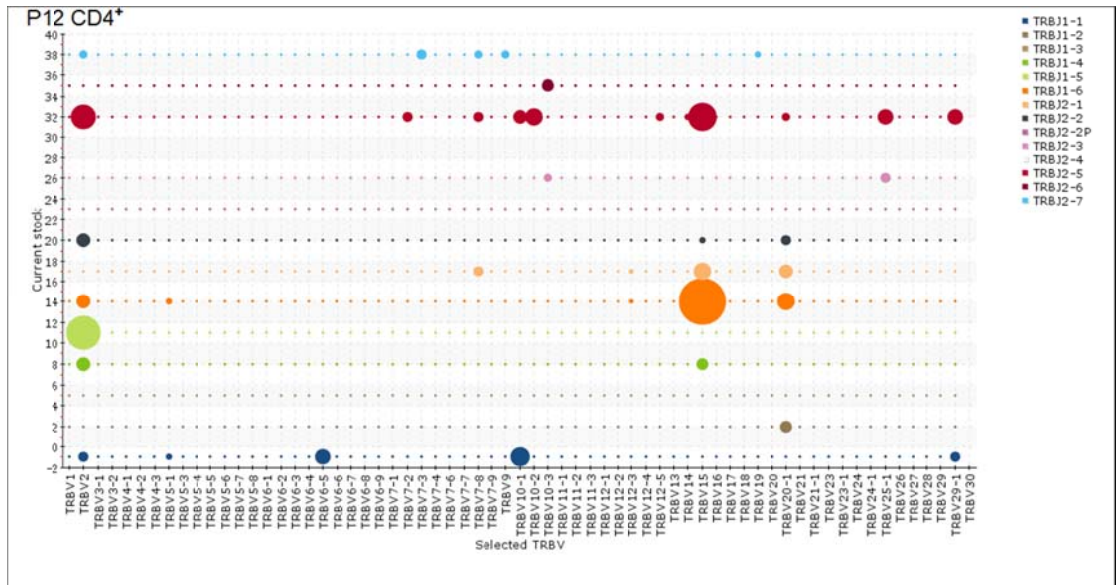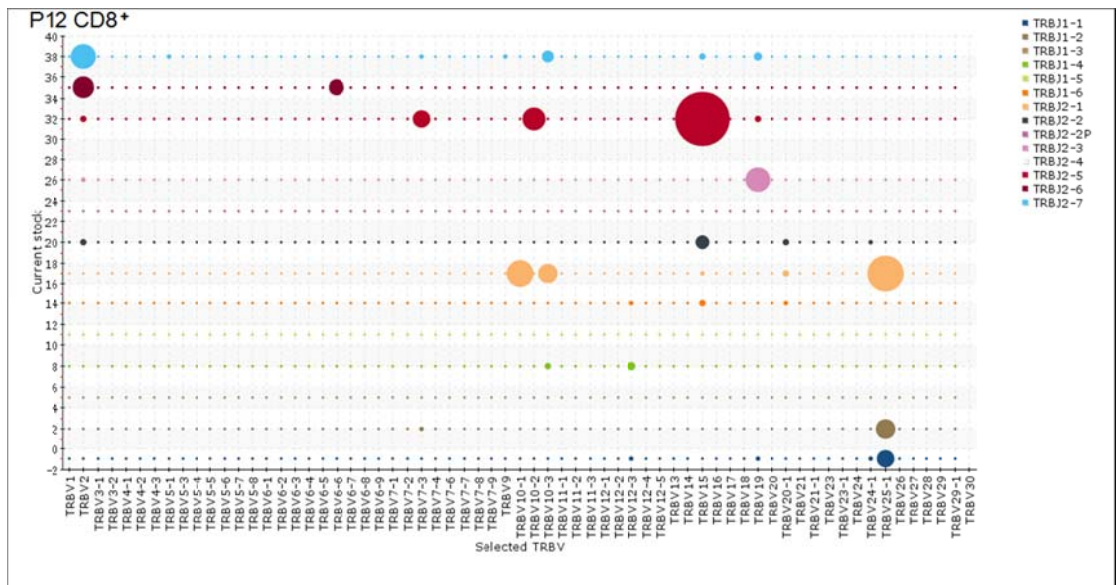

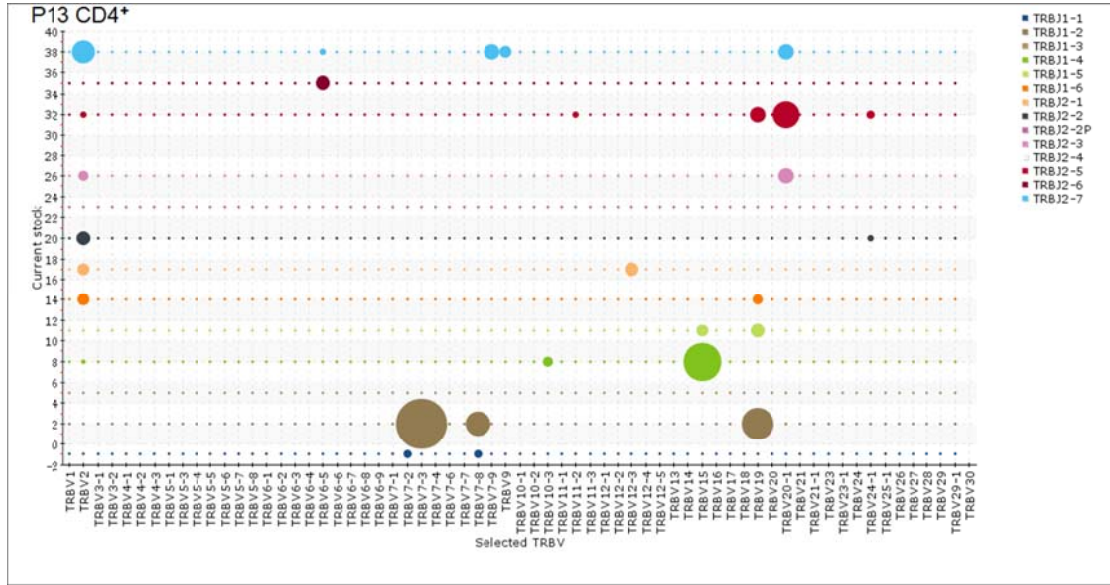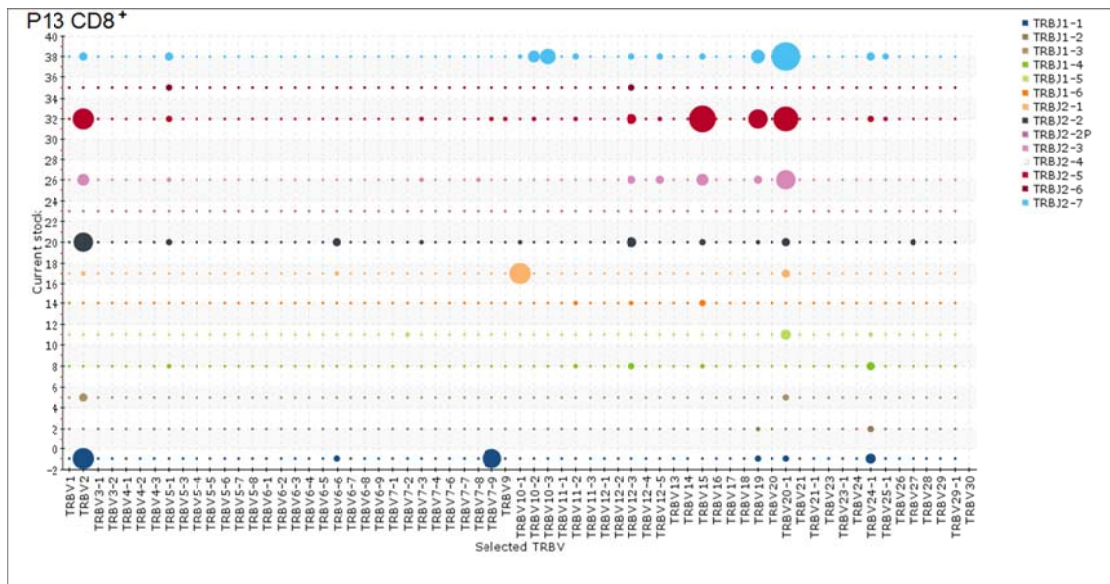

Supplement: Supplementary Figure S2 — The VJ combination usage patterns The VJ combination of individual samples is analyzed in the 2-dimensional plot. The X and Y axes list all possible V gene and J usages, respectively, while each point in the 2-dimensional space represents a unique VJ combination. The size of the sphere at each point represents the number of reads that match to a particular VJ combination. [file mmc2.pdf]

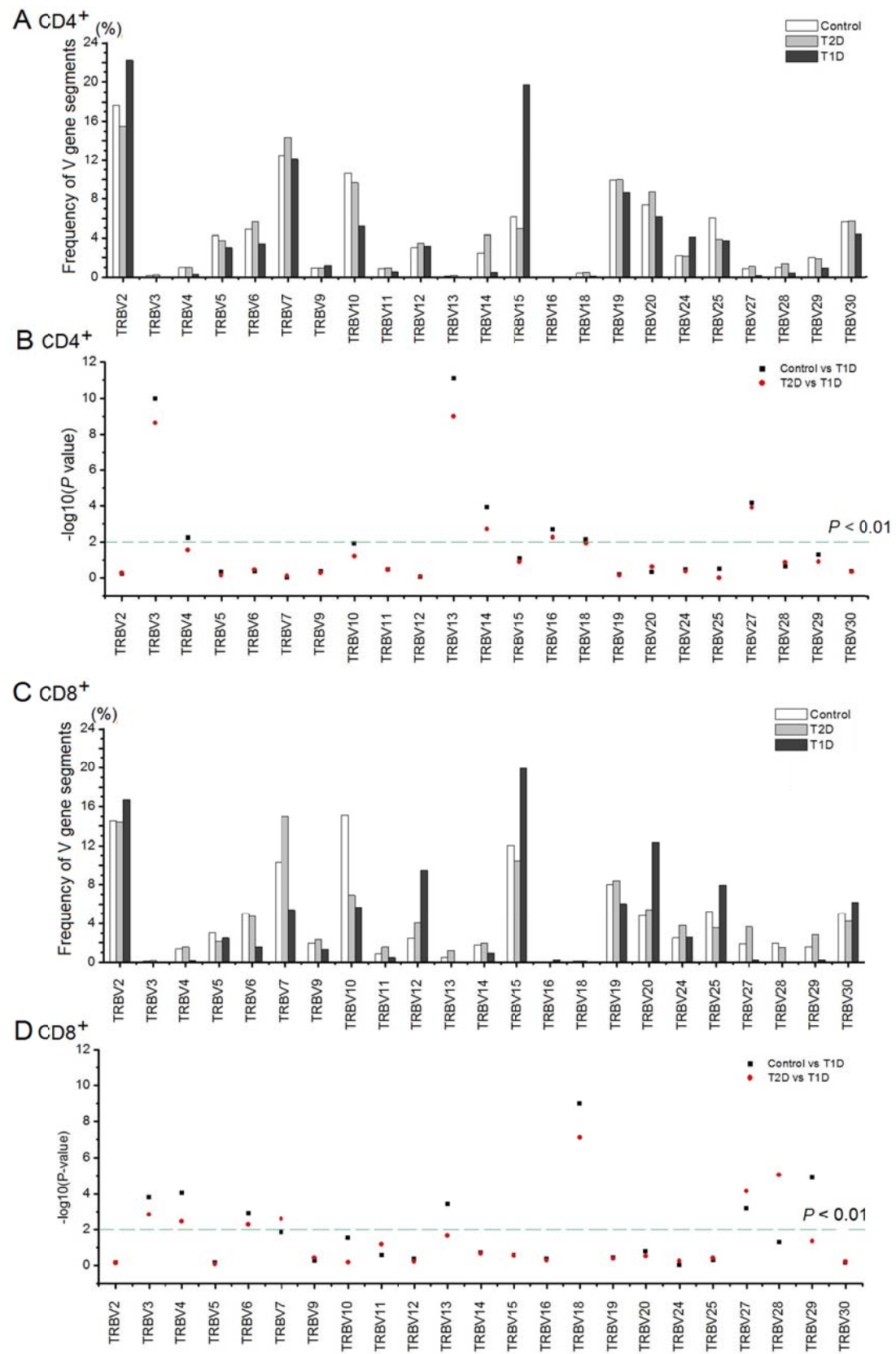

Supplement: Supplementary Figure S3 — TRBV gene usage patterns in CD4 and CD8 T cells The white, gray and black bars represent the control, T2D and T1D samples. The bar charts show the percentage of each TRBV used in CD4+ (A) and CD8+ T (C) cells from each group on average. To find out TRBV genes that are significantly highly expressed in CD4+ (B) and CD8+ T (D) cells from T1D samples, we calculated the P value by comparing control to T1D, and comparing T2D to T1D, using student t-test. The green dashed horizontal lines represent the significance level (P < 0.01). T1D, type 1 diabetes mellitus; T2D, type 2 diabetes mellitus. [file mmc3.pdf]

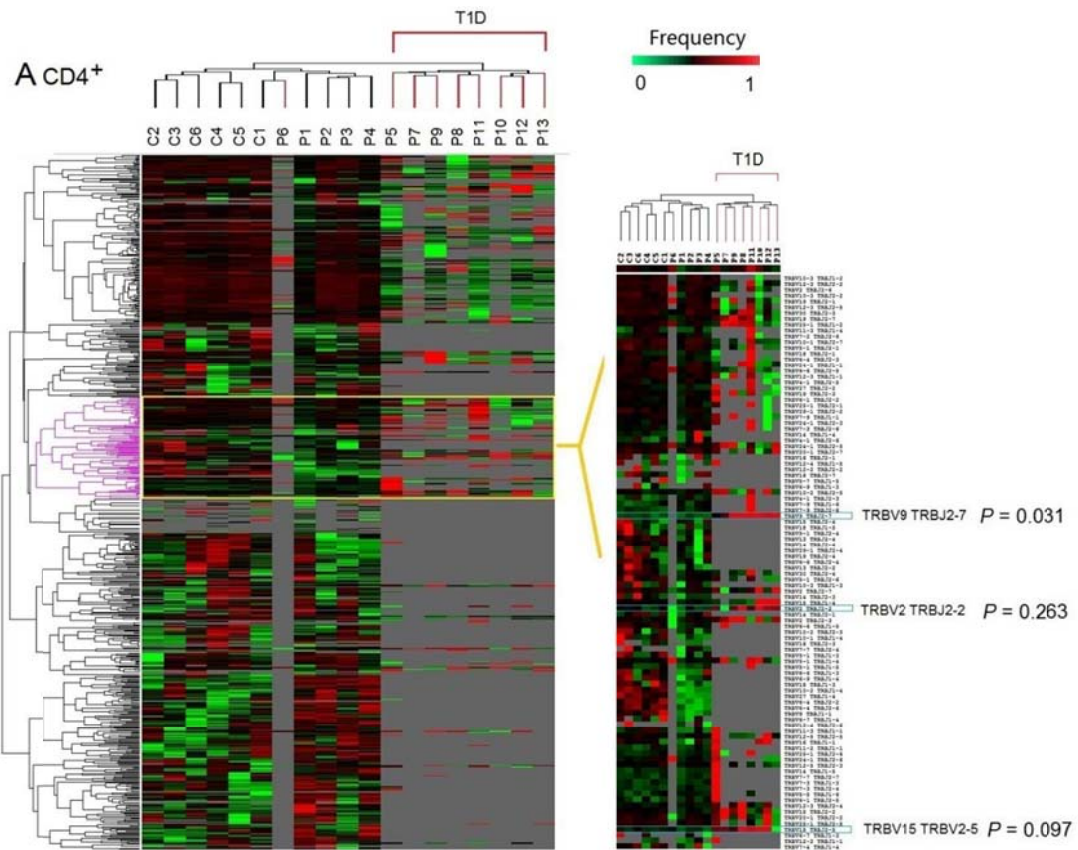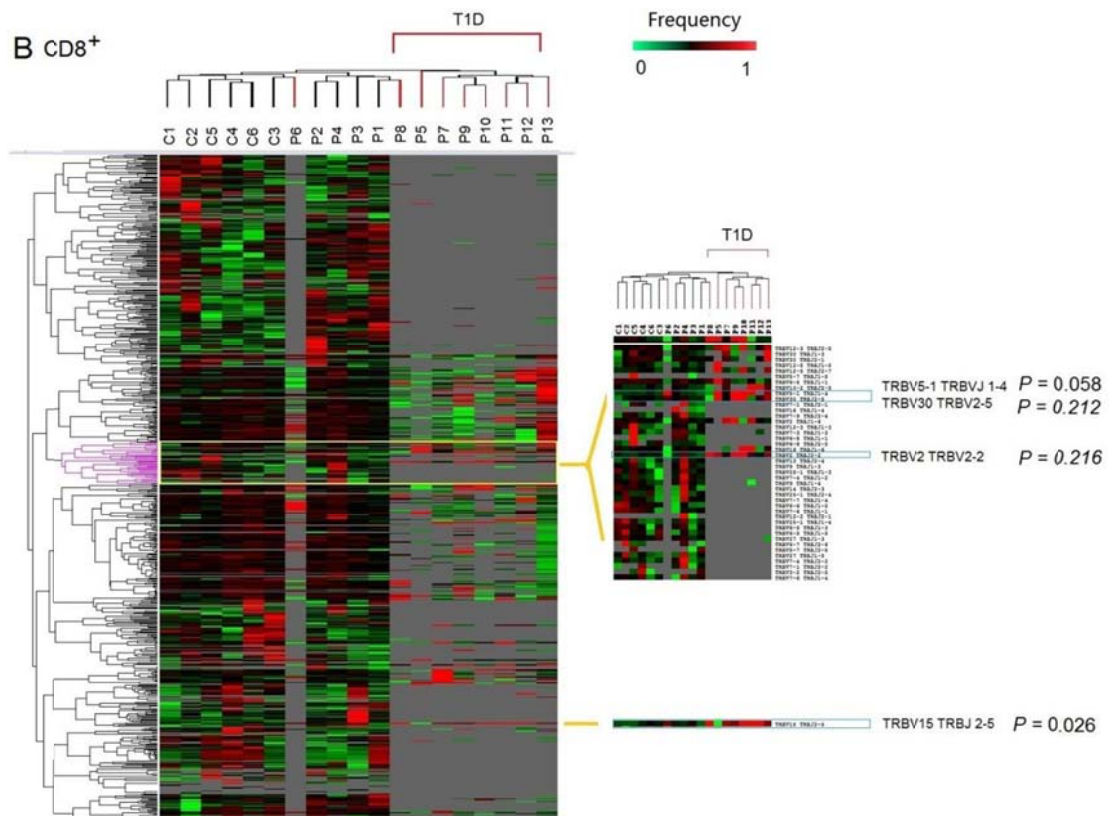

Supplement: Supplementary Figure S4 — VJ recombination clustering The T1D patients are grouped into a cluster, except sample P6. TRBV9–TRBJ2-7 combination is significantly more used in CD4+ cells of T1D patients (P = 0.031; A), while TRBV15–TRBJ2-5 is significantly more used in CD8+ cells of T1D patients (P = 0.026; B). Statistical analysis was performed using student t-test. The frequency of combinatorial VJ usage is color-coded in the figure. T1D, type 1 diabetes mellitus. [file mmc4.pdf]
