# Supplementary material for: T Cell Repertoire Diversity Is Decreased in Type 1 Diabetes Patients
Source: Genomics Proteomics Bioinformatics. 2016 Dec 24;14(6):338–48. doi: 10.1016/j.gpb.2016.10.003 (PMC5200939; doi:10.1016/j.gpb.2016.10.003)
Supplement: Supplementary Table S1 — High-resolution HLA genotyping for 6 T1D patients [file mmc6.docx]

**Table S1 High-resolution HLA genotyping for 6 T1D patients**

| Patient ID | HLA-A | HLA-B | HLA-DRB1 | HLA-C | HLA-DQB1 |
| --- | --- | --- | --- | --- | --- |
| P5 | *A*11:01:01*  *A*11:01:01* | *B*46:01*  *B*58:01* | *DRB1*03:01*  *DRB1*04:04:01* | *C*01:02*  *C*03:02* | *DQB1*02:01:01*  *DQB1*03:02:01* |
| P6 | *A*24:02*  *A*33:03:01* | *B*54:01:01*  *B*58:01:01* | *DRB1*03:01*  *DRB1*04:04:01* | *C*03:02*  *C*08:01:01* | *DQB1*02:01:01*  *DQB1*03:02:01* |
| P7 | *A*24:02*  *A*24:02* | *B*15:01:01*  *B*58:01:01* | *DRB1*03:01*  *DRB1*09:01:02* | *C*01:02*  *C*03:02* | *DQB1*02:01:01*  *DQB1*03:03:02* |
| P8 | *A*32:01:01*  *A*33:03:01* | *B*51:07:01*  *B*58:01:01* | *DRB1*01:01:01*  *DRB1*03:01* | *C*03:02*  *C*14:02:01* | *DQB1*02:01:01*  *DQB1*05:01:01* |
| P10 | *A*02:06:01*  *A*33:03:01* | *B*15:02:01*  *B*58:01:01* | *DRB1*03:01*  *DRB1*12:02* | *C*03:02*  *C*08:01:01* | *DQB1*02:01:01*  *DQB1*03:01:01* |
| P12 | *A*02:07:01*  *A*24:02* | *B*46:01*  *B*52:01* | *DRB1*09:01:02*  *DRB1*15:02:01* | *C*01:02*  *C*12:02:02* | *DQB1*03:03:02*  *DQB1*06:01:01* |

*Note*: HLA genotyping was performed for 5 major HLA types. There are 456 different HLA-A*02 proteins in humans; therefore the allele name could be *HLA-A*02:01* to *HLA-A*02:456*. These genes can be further separated by genetic screening. HLA, human leukocyte antigen; T1D, type 1 diabetes mellitus; TCR, T cell receptor.
