# Supplementary material for: T Cell Repertoire Diversity Is Decreased in Type 1 Diabetes Patients
Source: Genomics Proteomics Bioinformatics. 2016 Dec 24;14(6):338–48. doi: 10.1016/j.gpb.2016.10.003 (PMC5200939; doi:10.1016/j.gpb.2016.10.003)
Supplement: Supplementary Table S2 — Clinical information for subjects included [file mmc7.docx]

**Table S2 Clinical information for subjects included**

| Subject | ID | Age (year) | Sex | Glucose (mM) | C peptide (µg/l) | Age of first diagnosis | Treatment | Note | Native place |
| --- | --- | --- | --- | --- | --- | --- | --- | --- | --- |
| T1D patients | P5 | 23 | F | 26.70 | 0.29 | 19 | Insulin | NA | Guangdong |
|  | P6 | 10 | F | 25.13 | 0.13 | 8 | Insulin | DKA | Hunan |
|  | P7 | 13 | F | 33.60 | 0.10 | 13 | Insulin | DKA | Guangdong |
|  | P8 | 30 | M | 23.60 | 0.27 | 27 | Insulin | DKA, hyperthyreosis | Guangdong |
|  | P9 | 12 | F | 38.55 | < 0.05 | Unknown | Insulin | DKA | Guangdong |
|  | P10 | 23 | F | 22.56 | < 0.05 | 20 | Insulin | NA | Guangdong |
|  | P11 | 59 | F | 7.70 | < 0.81 | 56 | Insulin | NA | Guangdong |
|  | P12 | 10 | F | 11.10 | < 0.05 | 9 | Insulin | NA | Guangdong |
|  | P13 | 10 | M | 28.20 | < 0.05 | 10 | Insulin | NA | Guangdong |
| T2D patients | P1 | 26 | M | 12.70 | 0.29 | 20 | Oral medication | NA | Guangdong |
|  | P2 | 60 | M | 13.00 | 1.29 | 44 | Oral medication; insulin, | Diabetic foot | Guangdong |
|  | P3 | 71 | F | 7.90 | 8.21 | 60 | Oral medication | NA | Guangdong |
|  | P4 | 59 | F | 28.60 | 2.30 | 59 | Oral medications | NA | Guangdong |
| Controls | C1 | 29 | M |  |  |  |  |  | Guangdong |
|  | C2 | 25 | F |  |  |  |  |  | Liaoning |
|  | C3 | 25 | M |  |  |  |  |  | Jiangsu |
|  | C4 | 28 | F |  |  |  |  |  | Guangdong |
|  | C5 | 24 | F |  |  |  |  |  | Guangdong |
|  | C6 | 32 | M |  |  |  |  |  | Guangdong |

*Note*: T1D, type 1 diabetes mellitus; T2D, type 2 diabetes mellitus; NA, no other symptom observed; DKA, diabetic ketoacidosis.
