# Supplementary material for: T Cell Repertoire Diversity Is Decreased in Type 1 Diabetes Patients
Source: Genomics Proteomics Bioinformatics. 2016 Dec 24;14(6):338–48. doi: 10.1016/j.gpb.2016.10.003 (PMC5200939; doi:10.1016/j.gpb.2016.10.003)
Supplement: Supplementary Table S3 — Primer sequences for amplification of human TCR CDR3 [file mmc8.docx]

**Table S3 Primer sequences for amplification of human *TCR CDR3***

| Primer name | Primer sequence (5′–3′) |
| --- | --- |
| Forward primers | |
| TRBV2 | CAGACGTGTGCTCTTCCGATCTAGATTTCACTCTGAAGATCCGGTCCAC |
| TRBV9 | CAGACGTGTGCTCTTCCGATCTAGCCTGACTTGCACTCTGAACTAAACCT |
| TRBV14 | CAGACGTGTGCTCTTCCGATCTAGGGAGGGACGTATTCTACTCTGAAGG |
| TRBV15 | CAGACGTGTGCTCTTCCGATCTAGTTCTTGACATCCGCTCACCAGG |
| TRBV19 | CAGACGTGTGCTCTTCCGATCTAGTCCTTTCCTCTCACTGTGACATCGG |
| TRBV3-1 | CAGACGTGTGCTCTTCCGATCTAGAAACAGTTCCAAATCGMTTCTCAC |
| TRBV4-1/2/3 | CAGACGTGTGCTCTTCCGATCTAGCAAGTCGCTTCTCACCTGAATG |
| TRBV5-1 | CAGACGTGTGCTCTTCCGATCTAGGCCAGTTCTCTAACTCTCGCTCT |
| TRBV5-4/5/6/8 | CAGACGTGTGCTCTTCCGATCTAGTCAGGTCGCCAGTTCCCTAAYTAT |
| TRBV6-1/2/3/5/8 | CAGACGTGTGCTCTTCCGATCTAGCAATGGCTACAATGTCTCYAGAT |
| TRBV6-4 | CAGACGTGTGCTCTTCCGATCTAGTGATGGTTATAGTGTCTCCAGAG |
| TRBV6-9 | CAGACGTGTGCTCTTCCGATCTAGCGATGGCTACAATGTATCCAGAT |
| TRBV6-6 | CAGACGTGTGCTCTTCCGATCTAGGAATGGCTACAACGTCTCCAGAT |
| TRBV7-2/4/6/7/8 | CAGACGTGTGCTCTTCCGATCTAGGGGATCCGTCTCCACTCTGAMGAT |
| TRBV7-3 | CAGACGTGTGCTCTTCCGATCTAGGGGATCCGTCTCTACTCTGAAGAT |
| TRBV7-9 | CAGACGTGTGCTCTTCCGATCTAGGGGATCTTTCTCCACCTTGGAGAT |
| TRBV10-1 | CAGACGTGTGCTCTTCCGATCTAGCCTCACTCTGGAGTCTGCTGCC |
| TRBV10-2/3 | CAGACGTGTGCTCTTCCGATCTAGCCTCACTCTGGAGTCMGCTACC |
| TRBV11-1/2/3 | CAGACGTGTGCTCTTCCGATCTAGGCAGAGAGGCTCAAAGGAGTAGACT |
| TRBV12-3/4 | CAGACGTGTGCTCTTCCGATCTAGATCGATTCTCAGCTAAGATGCCT |
| TRBV12-5 | CAGACGTGTGCTCTTCCGATCTAGATCGATTCTCAGCAGAGATGCCT |
| TRBV13 | CAGACGTGTGCTCTTCCGATCTAGTCGATTCTCAGCTCAACAGTTC |
| TRBV18 | CAGACGTGTGCTCTTCCGATCTAGTAGATGAGTCAGGAATGCCAAAG |
| TRBV20-1 | CAGACGTGTGCTCTTCCGATCTAGAACCATGCAAGCCTGACCTT |
| TRBV24-1 | CAGACGTGTGCTCTTCCGATCTAGCTCCCTGTCCCTAGAGTCTGCCAT |
| TRBV25-1 | CAGACGTGTGCTCTTCCGATCTAGGCCCTCACATACCTCTCAGTACCTC |
| TRBV27/28 | CAGACGTGTGCTCTTCCGATCTAGGGAGATGTTCCTGARGGGTACA |
| TRBV29-1 | CAGACGTGTGCTCTTCCGATCTAGAACTCTGACTGTGAGCAACATGAG |
| TRBV16 | CAGACGTGTGCTCTTCCGATCTAGCTGTAGCCTTGAGATCCAGGCTACGA |
| TRBV30 | CAGACGTGTGCTCTTCCGATCTAGCAGATCAGCTCTGAGGTGCCCCA |
| Reverse primers | |
| TRBJ1.1 | CTACACGACGCTCTTCCGATCTCTTACCTACAACTGTGAGTCTGGTG |
| TRBJ1.2 | CTACACGACGCTCTTCCGATCTCTTACCTACAACGGTTAACCTGGTC |
| TRBJ1.3 | CTACACGACGCTCTTCCGATCTCTTACCTACAACAGTGAGCCAACTT |
| TRBJ1.4 | CTACACGACGCTCTTCCGATCTCATACCCAAGACAGAGAGCTGGGTTC |
| TRBJ1.5 | CTACACGACGCTCTTCCGATCTCTTACCTAGGATGGAGAGTCGAGTC |
| TRBJ1.6 | CTACACGACGCTCTTCCGATCTCATACCTGTCACAGTGAGCCTG |
| TRBJ2.1 | CTACACGACGCTCTTCCGATCTCCTTCTTACCTAGCACGGTGA |
| TRBJ2.2 | CTACACGACGCTCTTCCGATCTCTTACCCAGTACGGTCAGCCT |
| TRBJ2.3 | CTACACGACGCTCTTCCGATCTCCGCTTACCGAGCACTGTCAG |
| TRBJ2.4 | CTACACGACGCTCTTCCGATCTCCAGCTTACCCAGCACTGAGA |
| TRBJ2.5 | CTACACGACGCTCTTCCGATCTCGAGCACCAGGAGCCGCGT |
| TRBJ2.6 | CTACACGACGCTCTTCCGATCTCTCGCCCAGCACGGTCAGCCT |
| TRBJ2.7 | CTACACGACGCTCTTCCGATCTCTTACCTGTGACCGTGAGCCTG |

*Note*: Human *TCRβ* sequences (GenBank accession No. NG_001333) were downloaded from IMGT. A relatively-conserved region upstream of *CDR3* was selected for putative forward primer region. A cluster of primers corresponding to the majority of the V gene sequence family was selected. Similarly, reverse primers corresponding to 13 types of the J gene family were designed. The forward and reverse primers were analyzed by Oligo 7.0 and MFEprimer 2.0 software for primer dimer and loop structures. Minor changes in the sequences were made for low-quality primers. TCR, T cell receptor; IMGT, ImMunoGeneTics; CDR3, complementarity-determining region 3.
