# Supplementary material for: T Cell Repertoire Diversity Is Decreased in Type 1 Diabetes Patients
Source: Genomics Proteomics Bioinformatics. 2016 Dec 24;14(6):338–48. doi: 10.1016/j.gpb.2016.10.003 (PMC5200939; doi:10.1016/j.gpb.2016.10.003)
Supplement: Supplementary Table S4 — Summary of sequencing data [file mmc9.docx]

**Table S4 Summary of sequencing data**

| **Sample** | **ID** | **Total No. of TCR sequences** | | **Total No. of *CDR3* sequences** | | **No. of unique clonotypes** | | **Shannon entropy** | |
| --- | --- | --- | --- | --- | --- | --- | --- | --- | --- |
|  |  | **CD4^+^** | **CD8^+^** | **CD4^+^** | **CD8^+^** | **CD4^+^** | **CD8^+^** | **CD4^+^** | **CD8^+^** |
| T1D | P5 | 69,118 | 135,176 | 49,722 | 49,438 | 1453 | 479 | 0.432 | 0.166 |
|  | P6 | 4561 | 30,134 | 3492 | 23,037 | 144 | 600 | 0.455 | 0.422 |
|  | P7 | 6695 | 4519 | 5868 | 3193 | 193 | 304 | 0.321 | 0.491 |
|  | P8 | 47,820 | 117,007 | 39,775 | 56,951 | 524 | 548 | 0.330 | 0.175 |
|  | P9 | 115,098 | 117,375 | 80,929 | 90,281 | 1023 | 1158 | 0.258 | 0.313 |
|  | P10 | 143,334 | 42,955 | 93,325 | 37,186 | 701 | 372 | 0.230 | 0.202 |
|  | P11 | 63,109 | 24,888 | 44,618 | 15,027 | 1120 | 402 | 0.402 | 0.322 |
|  | P12 | 117,520 | 29,569 | 77,140 | 26,014 | 1337 | 625 | 0.328 | 0.330 |
|  | P13 | 41,956 | 1,073,150 | 38,563 | 765,452 | 609 | 7029 | 0.296 | 0.337 |
| T2D | P1 | 675,532 | 847,309 | 52,6416 | 677,530 | 183,715 | 161,645 | 0.859 | 0.698 |
|  | P2 | 653,706 | 765,762 | 50,8984 | 592,278 | 158,370 | 97,825 | 0.814 | 0.574 |
|  | P3 | 623,799 | 849,571 | 49,8960 | 655,766 | 193,702 | 124,882 | 0.871 | 0.649 |
|  | P4 | 840,351 | 853,186 | 64,8250 | 630,711 | 188,704 | 118,628 | 0.832 | 0.633 |
| Control | C1 | 639,614 | 643,557 | 44,3565 | 460,760 | 134,642 | 144,712 | 0.801 | 0.789 |
|  | C2 | 593,621 | 610,866 | 42,9686 | 405,479 | 157,341 | 113,588 | 0.857 | 0.760 |
|  | C3 | 600,427 | 657,111 | 43,6626 | 375,462 | 152,261 | 74,698 | 0.829 | 0.619 |
|  | C4 | 636,325 | 621,621 | 48,4738 | 463,035 | 165,366 | 132,884 | 0.846 | 0.636 |
|  | C5 | 556,765 | 537,576 | 36,814 | 350,211 | 126,478 | 111,415 | 0.846 | 0.809 |
|  | C6 | 475,839 | 540,742 | 29,1921 | 368,223 | 64,465 | 101,592 | 0.755 | 0.591 |

*Note*: The total number of TCR sequences refers to the number of identified TCR sequences after mapping raw reads against reference V/D/J sequences. The total number of CDR3 sequences refers to the number of sequences that can be annotated with CDR3 structure, according to IMGT definition. The number of unique clonotypes represents the number of T cell clones. Shannon entropy is used to evaluate the diversity of the CDR3 sequences. T1D, type 1 diabetes mellitus; T2D, type 2 diabetes mellitus; TCR, T cell receptor; IMGT, ImMunoGeneTics; CDR3, complementarity-determining region 3.
