# Supplementary material for: T Cell Repertoire Diversity Is Decreased in Type 1 Diabetes Patients
Source: Genomics Proteomics Bioinformatics. 2016 Dec 24;14(6):338–48. doi: 10.1016/j.gpb.2016.10.003 (PMC5200939; doi:10.1016/j.gpb.2016.10.003)
Supplement: Supplementary Figure S5 — Shared clones between T1D, T2D and control samples The complete list of shared HECs is shown in the heatmap. Orange and green represent HECs (⩾1% of total reads) and LECs (<1% of total reads), respectively, while gray represents sequences not found in the sample. HEC, highly-expanded clone; LEC, lowly-expanded clone; T1D, type 1 diabetes mellitus; T2D, type 2 diabetes mellitus. [file mmc5.pdf]

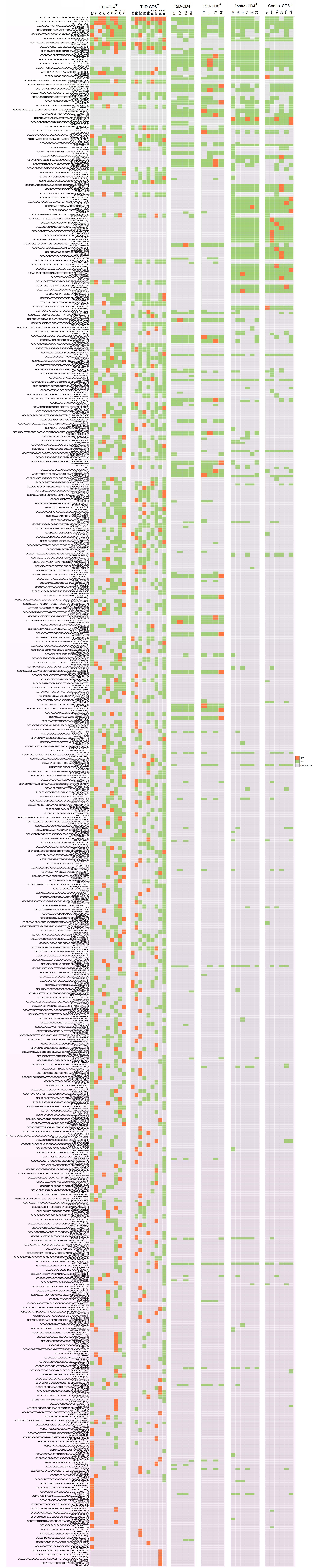

T1D-CD4+
T1D-CD8+
T2D-CD4+
T2D-CD8+
Control-CD4+
Control-CD8+
P5 P6 P7 P8 P9 P10 P11 P12 P13
P5 P6 P7 P8 P9 P10 P11 P12 P13
P1 P2 P3 P4
P1 P2 P3 P4
C1 C2 C3 C4 C5 C6
C1 C2 C3 C4 C5 C6
HEC
LEC
Non-detected
